# Characterization of a G-quadruplex from hepatitis B virus and its stabilization by binding TMPyP4, BRACO19 and PhenDC3

**DOI:** 10.1038/s41598-021-02689-y

**Published:** 2021-12-01

**Authors:** Orsolya Réka Molnár, András Végh, Judit Somkuti, László Smeller

**Affiliations:** 1 Department of Biophysics and Radiation Biology, Semmelweis University, Budapest, 1094 Hungary; 2 Department of Ophthalmology, Semmelweis University, Budapest, 1085 Hungary

**Keywords:** Biophysics, Molecular biology

## Abstract

Specific guanine rich nucleic acid sequences can form non-canonical structures, like the four stranded G-quadruplex (GQ). We studied the GQ-forming sequence (named HepB) found in the genome of the hepatitis B virus. Fluorescence-, infrared- and CD-spectroscopy were used. HepB shows a hybrid form in presence of K^+^, but Na^+^, Li^+^, and Rb^+^ induce parallel structure. Higher concentrations of metal ions increase the unfolding temperature, which was explained by a short thermodynamic calculation. Temperature stability of the GQ structure was determined for all these ions. Na^+^ has stronger stabilizing effect on HepB than K^+^, which is highly unusual. The transition temperatures were 56.6, 53.8, 58.5 and 54.4 °C for Na^+^, K^+^, Li^+^, and Rb^+^ respectively. Binding constants for Na^+^ and K^+^ were 10.2 mM and 7.1 mM respectively. Study of three ligands designed in cancer research for GQ targeting (TMPyP4, BRACO19 and PhenDC3) showed unequivocally their binding to HepB. Binding was proven by the increased stability of the bound form. The stabilization was higher than 20 °C for TMPyP4 and PhenDC3, while it was considerably lower for BRACO19. These results might have medical importance in the fight against the hepatitis B virus.

## Introduction

### G-quadruplex structure and its interactions

G-quadruplex is a non-canonical nucleic acid secondary structure that consists of two or three G-quartets each of them formed by four guanine nucleotides^1^. The quartet is stabilized by Hoogsteen-type base-pairing instead of the conventional Watson–Crick-type hydrogen bonds and monovalent cations such as sodium, potassium^2^. Regarding the orientation of the strands, they can appear in parallel, antiparallel or mixed forms^3^. Their actual form and stability is influenced by several factors including crowding conditions, osmolytes, temperature, pressure, ionic strength and ligands^4–11^. G-quadruplexes can interact in vitro and in vivo with various ions and molecules—this gives them their special biological significance. In vivo they take part in the regulation of gene expression, in vitro they are used in analytical biochemistry^12^.

Quadruplex structures were first identified in the telomere regions^13^ and promoter regions of oncogenes in the human genome that indicates their role in oncogenesis^14,15^. They can be found in the promoters of c-MYC, c-KIT, B-cell lymphoma protein-2 (BCL-2), vascular-endothelial growth factor (VEGF), platelet-derived growth factor (PDGF), retinoblastoma protein (RB), KRAS, hypoxia-induced factor-1 (HIF-1) and human telomeric reverse transcriptase (hTERT), for example^16–18^. G-quadruplexes form a mechanical obstacle for the replicase complex; thus their stabilization can provide anticancer effect^19^. As quadruplexes interact with positively charged ions and polyaromatic molecules, they are the targets for platinum ion containing drugs like cisplatin or carboplatin^20,21^.

When GQ-prone sequences were found in the telomere region and in oncogene sequences, they became targets for cancer research^22,23^. Several molecules were developed and characterized to bind to the telomere GQ and other GQs. The most widely known among these ligands is the TMPyP4 (meso-5,10,15,20-Tetrakis-(*N*-methyl-4-pyridyl) porphine) which binds to the telomere GQ. It was proven that its binding inhibits the telomerase activity. Telomerase elongates the telomere of the tumor cells, preventing cell death. Although TMPyP4 also binds to the double helices, its high affinity binding to telomere GQ made TMPyP4 a promising candidate for the cancer treatment^24^.

The trisubstituted acridine compound BRACO19 (*N*,*N*′-(9-(4-(Dimethylamino)phenylamino)acridine-3,6-diyl)bis(3-(pyrrolidin-1-yl)propanamide) hydrochloride) was shown to inhibit telomerase activity in cells and tumor xenografts^22^, and it has also been proven to have antitumor activity^25^.

PhenDC3 (3,3′-[1,10-Phenanthroline-2,9-diylbis(carbonylimino)]bis[1-methylquinolinium] 1,1,1-trifluoromethanesulfonate (1:2) is a highly promising molecule that was reported to bind specifically to GQs, such as the one derived from the c-myc promoter. Due to its high-affinity binding, it has been also used in assays to indicate formation of G-quadruplex^26^.

G-quadruplex structures are to be found in any genome and this statement applies to pathogens too from viruses through bacteria to protozoa. Quadruplexes act as cis-regulatory domains in nucleic acids controlling gene expression that modulates translation and can facilitate the microbe’s evasion from the immune system. This means that the pathogens can change their surface epitomes in order to evade the host’s immunity^27,28^. G-quadruplexes can control recombination-mediated antigenic variation in bacteria, for example *Borrelia burgdorferi* (cause of Lyme disease), *Treponema pallidum* (cause of syphilis), *Mycobacterium tuberculosis*, *Neisseria meningitidis* and gonorrhoeae^27,28^. Quadruplex structures have been detected in the genome of parasites such as *Plasmodium falciparum* (cause of malaria) and *Trypanosoma brucei* (cause of sleeping sickness) but their role still remains enigmatic. It has been shown that quadruplex-stabilizing drugs that accumulate in blood suppress the reproduction of such protozoa. Ironically, these molecules (e.g. quarfloxin) have failed as anticancer drugs due to their poor pharmacokinetics and distribution^27–30^. G-quadruplexes are present in viral genome in both DNA and RNA viruses and they take part in the key steps of viral infection of the cell from replication to encapsidation^31^. They have been found in many viruses: the members of Herpesviridae, such as herpes simplex virus 1 (HSV-1), Epstein-Barr virus (EBV), human herpesvirus 6 (HHV6) and Kaposi’s sarcoma associated herpesvirus (KSHV) as well as several others like human papillomavirus (HPV), Zika virus (ZIKV), Ebola virus (EBOV), severe acute respiratory syndrome coronavirus (SARS-CoV), simian virus 40 (SV40) and Hepatitis viruses A, B and C (HAV, HBV, HCV)^32–34^.

### Hepatitis B virus (HBV)

HBV is the most frequent chronic viral infection. Estimated 2 billion people have contacted the virus and currently about 350–400 million people are infected^35^. In 2010, HBV infection was the tenth leading cause of death globally^36^.

HBV is the only member of the Hepadnaviridae family that can infect human cells; it contains partially double-stranded DNA (dsDNA) and possesses reverse transcriptase (RT) enzyme activity. It has a small circular DNA genome with the length of approximately 3.2 kilobase pairs^35,37^. HBV has four overlapping reading frames that encode seven proteins (including S for surface or envelope gene, C for the core gene and P for the polymerase gene)^37,38^. It is classified into ten genotypes (A-J) based on nucleotide variation^36,37^.

Lavezzo et al.^31^ analysed genomes of several viruses. For the HBV the highest G-score was associated to the GGC TGG GGC TTG GTC ATG GGC CAT CAG (NC_003977.2:1204..1230 (+ strand)) sequence. This sequence can be found in the coding region of the polymerase protein. This paper focuses on the stability of this GQ and on the targeting of this GQ with ligands developed for cancer therapy: TMPyP4, BRACO19 and PhenDC3. These ligands were developed with the aim to stabilize the telomere and oncogene promoter GQs in order to reduce the cancerous development. We investigated whether these ligands also influence the viral HepB GQ.

## Material and methods

### Materials

The oligonucleotide GGC TGG GGC TTG GTC ATG GGC CAT CAG was named HepB and purchased from IDT (NY, USA) and Sigma-Aldrich Kft (Hungary). The oligo labeled with the FRET pair FAM and TAMRA (HepB_FRET) was also purchased from the same sources. The oligos were obtained from the manufacturers in lyophilized form.

For the fluorescence experiments the HepB_FRET samples were first dissolved in MilliQ water in a concentration of 100 μM, according to the suggestion of IDT. This stock solution was kept frozen and diluted with an appropriate buffer during sample preparation. The final concentration of the oligos in the samples was 1 μM, unless stated otherwise. For the heating experiments 100 mM K-phosphate and Na-phosphate buffers (pH 7.4) were used, because of their insensitivity to temperature. For experiments with Li^+^ and Rb^+^ Tris buffer was used. In this case the pH of the solution was 7.4 at the transition temperature, taking into account the − 0.024 pH unit/°C drift value (calculated from the Sigma product information page). The ion titration experiments were performed in 1 mM Tris–HCl buffer (pH 7.4). TMPyP4 was purchased EMD (USA) and ChemCruz (Dallas, USA), BRACO19 and PhenDC3 were purchased from Sigma-Aldrich Kft. (Hungary).

In the infrared experiments much higher concentration of HepB was required. The oligo was dissolved in D_2_O based Bis–Tris buffer (100 mM, pD 7.4) in a concentration of 20 mg/ml. The metal ion concentrations were 100 mM.

For the CD experiments the same buffers were used as for the fluorescence experiments. The concentration of the oligo was 12 µM.

All chemicals not specified above differently were purchased from Sigma-Aldrich.

### Spectroscopy

Fluorescence experiments were performed as described earlier in detail^39,40^. Two spectrometers were used: Fluorolog-FL3 fluorimeter (Horiba Jobin Yvon, France) was used mainly for the temperature scans. It was equipped with a programmable temperature controlled cell (DI instruments, Hungary). Some of the titration experiments were performed on the FLS 980 of Edinburgh Instruments (UK). The cell was temperature controlled in this case, too (QNW Luna 40, Quantum Nortwest, WA). An HH802U thermometer and the corresponding software from Omega were used in both spectrometers to record the temperature by a thermocouple directly in the cuvette (Omega, USA).

In the absorption spectroscopic measurements the TMPyP4 concentration was determined by the absorption of the Soret band using the extinction coefficient of 2.26 × 10^5^ M^−1^ cm^−1^^41^.

FTIR spectra were measured with a Bruker Vertex 80v spectrometer. 256 spectra were averaged at 2 cm^−1^ resolution. D_2_O buffer was used as solvent, in order to avoid the large absorption band of water around 1640 cm^−1^. The samples were measured in a temperature controlled diamond anvil cell (Diacell, UK) to reduce the sample volume.

CD spectra were measured using a JASCO 1500 CD/LD spectrometer in CD mode. The sample was injected into a cuvette with 1 mm path length. Three spectra recorded in 1 nm steps were averaged for each sample. The average spectra were smoothed by a boxcar function. These spectra were recorded at room temperature.

Absorption spectra were measured by a Cary4 UV–Vis spectrometer. Spectra were smoothed with 9-point Savitzky-Golay filter^42^. Spectral parameters, like peak position and amplitude were evaluated with the PIW program using the Savitzky–Golay peak finding algorithm^42,43^.

### Dissociation constant

The dissociation constant was determined from the fit of the data. The definition of the dissociation constant is:

1 $${\text{K}}_{{\text{d}}} = \left[ {\text{L}} \right]\left[ {\text{G}} \right]/\left[ {{\text{GL}}} \right],$$

where [L], [G] and [GL] are the concentrations of the free ligand, free GQ and the bound GQ-ligand complex respectively. Solution of this equation for [G] gives the following equation:

2 $$\left[\text{G}\right]=-A+\sqrt{{{A}^{2}}+{{c}_{G}}{{K}_{d}}},\,\text{where }A=({c}_{\text{L}}-{c}_{\text{G}}+{K}_{\text{d}})/2.$$

Here *c*_L_ and *c*_G_ denote the total concentrations of the ligand and the GQ. We used this equation for the determination of *K*_d_, since *c*_L_ and *c*_G_ both change during the titration experiment. Similarly:

3 $$\left[\text{L}\right]=-B+\sqrt{{B}^{2}+{c}_{L}{K}_{d}, }\,\text{where }B=({c}_{\text{G}}-{c}_{\text{L}}-{K}_{\text{d}})/2 .$$

In the metal ion binding experiments, the labeled oligo gave the measured fluorescent signal. In this case the donor fluorescence was used to measure the free (unfolded) oligo concentration. [G] was obtained from the fluorescence signal in the following way:

4 $$\left[ {\text{G}} \right] = \, \left( {F - F_{{{\text{bound}}}} } \right)/a.$$

Similarly, if the ligand (TMPyP4, BRACO19 and PhenDC3) did have fluorescence signal, we used:

5 $$\left[ {\text{L}} \right] = \, \left( {F - F_{{{\text{bound}}}} } \right)/a,$$

where *F* means the fluorescence intensity at the given concentration, *F*_bound_ is the intensity in case of complete binding and *a* is a fitted parameter. In the metal ion titration experiments the ion concentration was increased, while the oligo concentration decreased only due to dilution of the sample. In the ligand binding experiments the GQ concentration was increased, while the total ligand concentration of the solution changed only due to the dilution. It has to be mentioned that the fitting is mathematically ill-conditioned, if the resulting *K*_d_ is smaller than c_L_. In this case the *K*_d_ value was accepted as the highest estimate of the dissociation constant.

### Temperature stability experiments

The unfolding temperature of the GQ was determined from the fit of the following equation:

6 $$y(T) = a + bT + \frac{\Delta a + \Delta bT}{{1 + \exp \left( {\frac{\Delta H}{R}\left( {\frac{1}{T} - \frac{1}{{T_{m} }}} \right)} \right)}}.$$

Here, *y* is the physical parameter to be fitted (e.g. fluorescence intensity, or ratio of fluorescence intensities at two different wavelengths), a and b are the parameters describing the linear dependence of y(T) below the transition, *T* is the thermodynamic temperature, Δ*a* and Δ*b* are the changes of *a* and *b* during the transition, Δ*H* is the enthalpy change, *R* is the universal gas constant, and *T*_m_ is the transition midpoint.

## Results and discussion

### Characterization of HepB structure and stability by FRET and FTIR measurements

**Figure 1:**
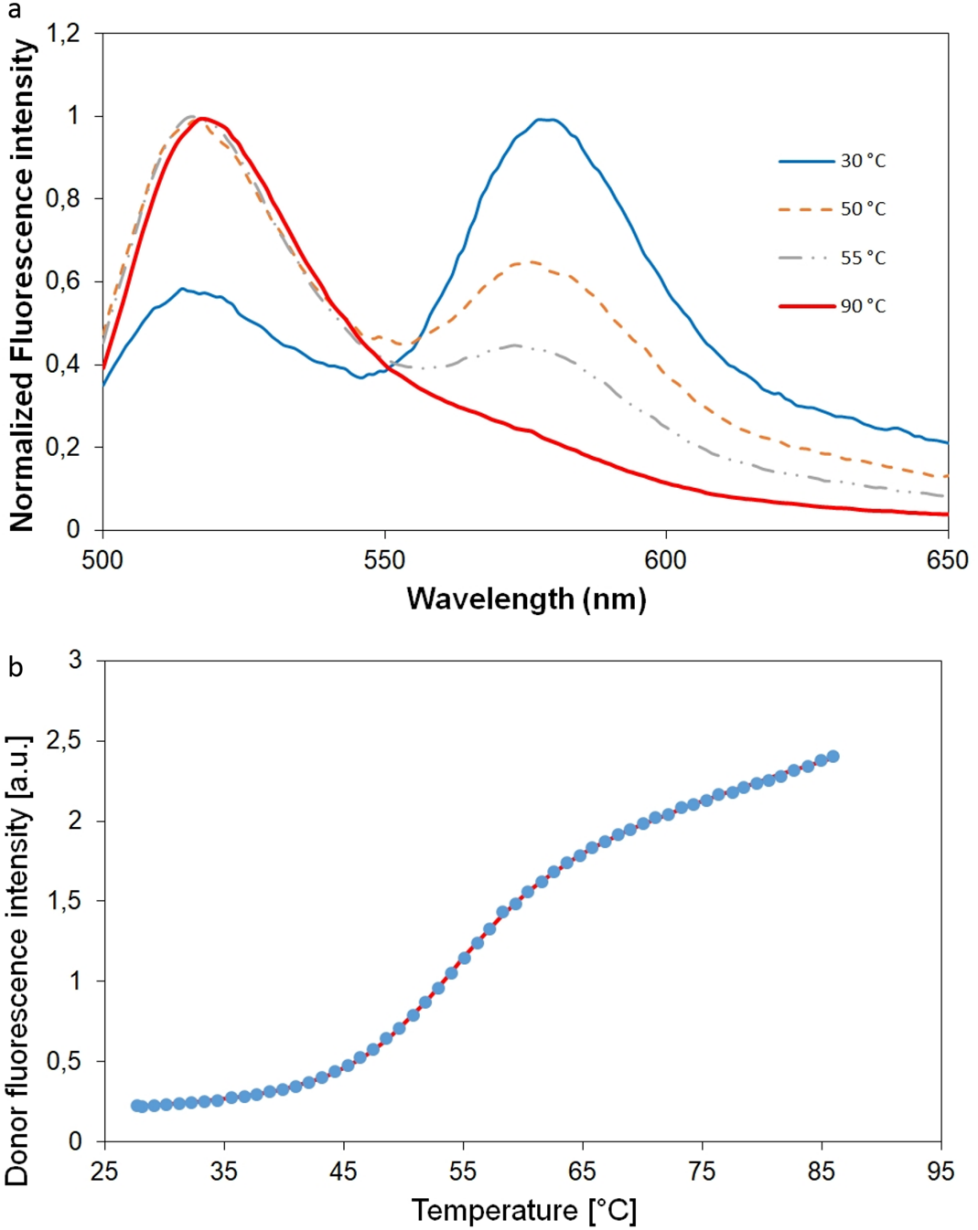
(**a**) Fluorescence spectra of HepB labeled by a FRET pair of FAM and TAMRA in K-phosphate buffer containing 140 mM K^+^ ion at few selected temperatures. (**b**) The donor fluorescence intensity vs. temperature. The line represents the fitting of Eq. (6).

FRET has been proven to be suitable for studying GQ conformational changes^6,44^. Figure 1a and Supplementary Fig. S1 shows the fluorescence spectra of the HepB_FRET at selected temperatures in presence of 140 mM K^+^ ion. Increase of the donor intensity relative to the acceptor intensity shows the loss of the fluorescence energy transfer due to increased distance between the fluorophores. The increase of the distance indicates unfolding of the GQ structure. The donor intensity vs. temperature is plotted in Fig. 1b. Fitting the Eq. (6) results in a transition temperature of *T*_m_ = 53.1 °C. The measured temperature is comparable with the typical values measured for two-quartet GQs. One of the most known two-quartet GQ is the thrombin binding aptamer (TBA). Sugimoto’s lab studied this GQ extensively using optical spectroscopic techniques. Their measured *T*_m_ value in KCl is 51.2 °C, which is very close to our results. Although the stability of GQs is also influenced by the composition and length of the loops^45,46^ typical unfolding temperature of two staged GQs lies around 50°. In contrary, 3-quartet GQs like Htel, c-MYC or KIT1 have an unfolding point higher than 60 °C in presence of K^+^ ions^39,40^.

Observing the sequence of HepB we can hypothesize a two-quartet structure, since the shortest guanine repeats contain only two guanine bases. Although there is a GGGG repeat present and the last guanine in the sequence can fold back, the formation of a complete third G-quartet is not possible.

This GQ has loops with a various length from two to six bases. Effect of loop length on the stability of GQs was systematically investigated by Guedin et al.^45^ They investigated the G_3_T_x_G_3_T_y_G_3_T_z_G_3_ sequence, where x,y,z, are integer numbers indicating the loop lengths. They found a clear destabilizing effect of loops larger than 3–4 bases. In presence of Na^+^ the *T*_m_ values were lower compared to those in presence of K^+^, and the destabilizing effect is also higher in case of K^+^.

As mentioned previously, GQs form only in presence of cations. HepB adopts the GQ structure in presence of several stabilizing metal ions. We compare stabilizing effect of four ions and ask the question: to what extent other monovalent cations stabilize the folded form of the oligo. It is a common belief that K^+^ or Na^+^ ions are able to stabilize the folded form, and K^+^ leads to a more stable structure. This was measured in case of several inter and intramolecular GQs^47–50^. Li^+^ is believed to be too small to stabilize the structure^51^, while stabilizing effect of Rb^+^ ion is between that of Na^+^ and K^+^^48^. Our results however show an interesting deviation from this common trend. Table 1. shows the unfolding temperature (*T*_m_) of HepB_FRET in presence of 140 mM of monovalent ions: Na^+^, K^+^ Li^+^ and Rb^+^. As it can be seen all the ions are able to stabilize the GQ structure at 140 mM concentration. Higher concentration increases the unfolding temperature both in the case of Na^+^ and K^+^, which meets our expectations. The surprising result is the reversal of the effects of K^+^ and Na^+^ compared to the expectations. Contrary to other oligos, in case of HepB_FRET Na^+^ seems to be a more effective stabilizer than K^+^. The transition temperatures of Na-stabilized GQs are higher by 2–3 °C. This difference can be observed in the whole concentration range we studied. Such a behavior is unique as far as we know. As mentioned above all the literature data report higher T_m_ for K^+^ stabilized GQs than for Na^+^ stabilized ones. In our earlier works we also investigated a series of GQs^39,40^ and K^+^ was the more potent stabilizer in all cases. We obtained more than 15 °C higher *T*_m_ in KCl for our above mentioned oligos. McGregor’s lab also reported a 10 °C stabilization of human telomere GQ in K^+^ compared to Na^+^^52^. Several other papers also reported higher stability in presence of K^+^^45,53^. So according to the literature a higher *T*_m_ value in KCl is a general phenomenon and HepB is the first example that violates this rule.

**Table 1:** Transition temperature (*T*_m_) values of HepB_FRET in presence of 140 mM ions.

Ion type	*T*_m_ (°C)
Na^+^	56.6 ± 0.4
Li^+^	58.5 ± 0.4
Rb^+^	54.4 ± 0.4
K^+^	53.8 ± 0.3*

*Average of two parallel measurements.

Li^+^ is known to be too small to stabilize the GQ structure, but in this case the stabilizing effect of Li^+^ is even higher than that of Na^+^ and K^+^. We hypothesize that this might be the result of the relatively large loops, which allow a more relaxed core structure.

Rb^+^ has the biggest ionic radius of 152 pm. Although its dimension is considerably larger than that of Na^+^ (95 pm) and K^+^ (133 pm), Rb^+^ was also proven to be capable of stabilizing some of the GQs^54^. According to the literature its stabilizing effect is comparable with that of Na^+^. In case of HepB it builds a GQ with similar stability as K^+^, but weaker than that of Na^+^.

**Figure 2:**
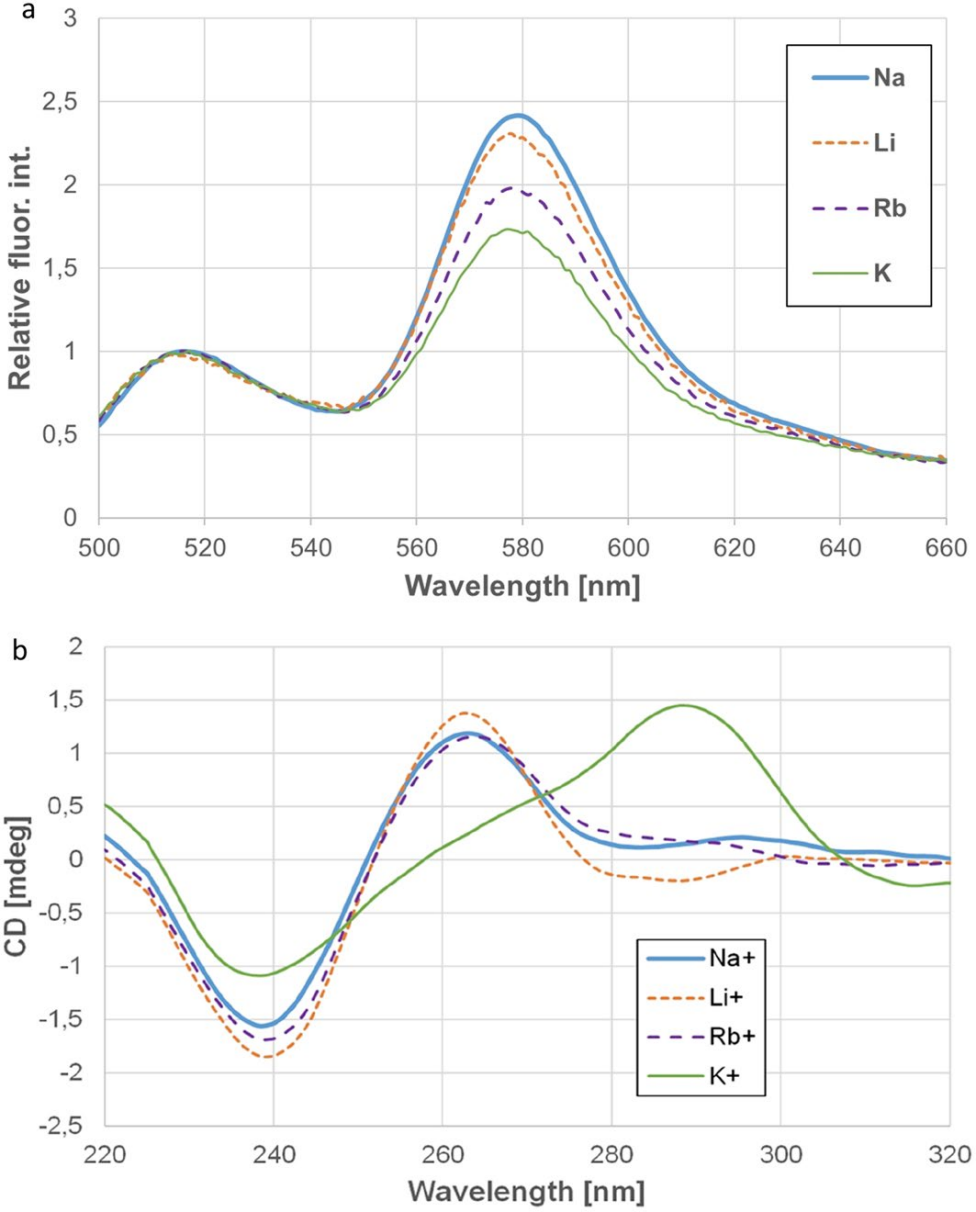
(**a**) Fluorescence spectra of HepB_FRET in presence of Na^+^, K^+^, Li^+^ and Rb^+^ at 30 °C. These spectra are normalized to the donor intensity around 518 nm. (**b**) CD spectra of HepB at room temperature in presence of the same ions.

Figure 2a shows the normalized fluorescence spectra of HepB_FRET at 30 °C in case of different cations used in this study. The spectra are normalized to the donor emission intensity. In this case the intensity of the acceptor emission shows the efficiency of the energy transfer. The higher the acceptor intensity is, the smaller is the donor–acceptor distance. Since the FRET efficiency depends on the distance very strongly, the observed 40% variation in the acceptor intensity indicates a very slight difference in the distance, i.e. very small distortion of the structure. The general trend of higher acceptor intensity in case of higher temperature stability can however be observed for K^+^, Rb^+^ and Li^+^. This indicates a more compact structure in case of higher temperature stability. Na^+^ does not fit in this trend. The energy transfer is the most efficient in the presence of Na^+^ that suggests that the structure is the most tightly packed when it is stabilized by Na^+^.One can argue of course that different ions induce different structures, which can explain the alterations in the stability. It was reported that K^+^ stabilizes the parallel, while Na^+^ induces the antiparallel structures^49,55^. On the other hand, in case of Htel K^+^ stabilizes a hybrid structure, while Na^+^ prefers the antiparallel one^56^. In order to check if there are different structures formed in case of different cations, we performed CD experiments. As the CD results (Fig. 2b) show, Na^+^, Li^+^ and Rb^+^ induce the same structure, while the CD spectra indicate a different conformation in case of K^+^. The positive band around 260 nm accompanied with a negative band at 240 nm indicates a parallel structure in case of all cations except K^+^^57,58^. The CD spectrum of the sample with K^+^ shows a positive band at 290 nm, a shoulder at 270 nm and a negative band at 240 nm. This is indicative for the hybrid mixed parallel and antiparallel structure. Such hybrid structure was found in case of human telomere in presence of K^+^^59^. From our CD experiments we can conclude, that Na^+^ Li^+^ and Rb^+^ induces a parallel structure, while HepB shows a hybrid form in presence of K^+^.

**Figure 3:**
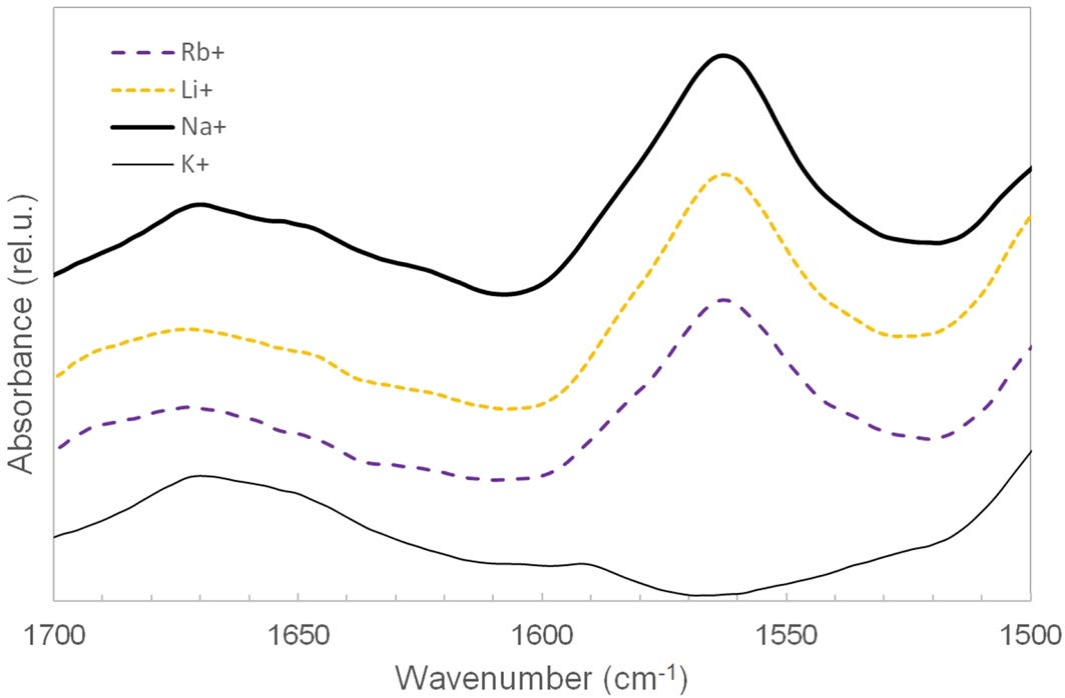
Infrared spectra of HepB in presence of 100 mM Na^+^, K^+^, Li^+^ and Rb^+^ at pD7.4 at 30 °C.

Infrared spectra of HepB in presence of different metal ions can be seen in Fig. 3. The band at 1672 cm^−1^ belongs to the C_6_=O_6_ vibration. The position of this band implies the presence of GQ structure^40^. The most prominent difference between the spectra is the appearance of the band at 1563 cm^−1^ in case of Na^+^, Li^+^ and Rb^+^. This vibration is absent in the K^+^ induced GQ form. The band can be assigned to C=N and C–N stretching not including the N_7_ atom^40,60^. This band was present in our previous experiments on Htel but in much lower amount^40^. This intense band seems therefore to be characteristic for the presence of the parallel structure.

**Figure 4:**
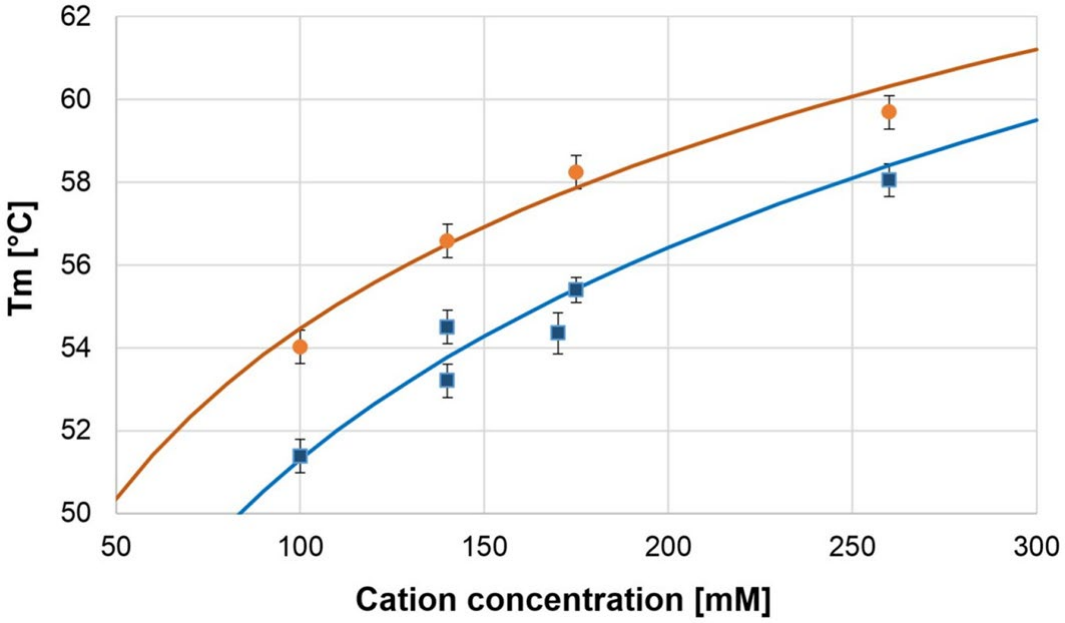
The unfolding temperature of HepB versus concentration of Na^+^ (filled circle) and K^+^ (filled square) ions. The fitted curves corresponding to the Eq. (10) are also plotted.

Effect of Na^+^ and K^+^ concentrations on the thermal stability of the GQ was studied in the range of 100 to 250 mM. Figure 4 shows the thermal stability vs. Na^+^ and K^+^ concentrations. The unfolding curves can be seen in Supplementary Figs. S2 and S3.

In order to understand how and why the transition midpoint depends on the concentration of the metal ions, a short thermodynamic calculation is necessary. The concentration dependence of *T*_m_ can be explained by considering the Gibbs free energy change of the unfolding: Δ*G*_u_ = *G*_ss_ − *G*_GQ_, where *G*_ss_ and *G*_GQ_ are the Gibbs free energies of the unfolded single stranded oligo, and the folded GQ respectively. Δ*G*_u_ can be written as:

7 $$\Delta G_{{\text{u}}} = \mu_{{{\text{ss}}}} \times \Delta {\text{v}}_{{{\text{ss}}}} + \mu_{{\text{K}}} \times \Delta {\text{v}}_{{\text{M}}} + \mu_{{{\text{GQ}}}} \Delta {\text{v}}_{{{\text{GQ}}}} ,$$

where *µ* and *ν* are the chemical potential and the amount of substance. M denotes the free metal ion Na^+^ or K^+^ in the solution.

Substituting *µ* = *µ*_0_ + *RT*⋅ln(*c*/*c*_0_) and Δ*ν* = Δ*ν*_M_ = Δ*ν*_ss_ =  − Δ*ν*_GQ_ we obtain:

8 $$\Delta G_{{\text{u}}} = \Delta {\text{v}} (\mu_{{{\text{ss}}0}} + \mu_{{{\text{M}}0}} - \mu_{{{\text{GQ}}0}} + RT \times {\text{ln}}((c_{{{\text{ss}}}} \times c_{{\text{M}}} )/(c_{{{\text{GQ}}}} \times c_{0} ))),$$

defining *µ*_00_ = *µ*_ss0_ + *µ*_M0_-*µ*_GQ0_ *ΔG*_u_ can be written as:

9 $$\Delta G_{{\text{u}}} = \Delta {\text{v}} (\mu_{00} + RT \times {\text{ln}}((c_{{{\text{ss}}}} \times c_{{\text{M}}} )/(c_{{{\text{GQ}}}} \times c_{0} ))).$$

At the transition temperature *T*_m_ the half of the oligos are folded: *c*_ss_ = *c*_GQ_ and *ΔG*_u_ = 0. Since the metal ions are present in large excess (*c*_M_ >  > *c*_G_ + *c*_ss_), *c*_M_ the concentration of free metal ions can be treated as equal to the total metal ion concentration of the solution. Substituting these values we obtain:

10 $${\text{T}}_{{\text{m}}} = - \mu_{00} /\left( {R \times {\text{ln}}\left( {c_{{{\text{Mtotal}}}} /c_{0} } \right)} \right).$$

The Na^+^ and K^+^ concentration dependence of the transition temperature was fitted with the above function, and we obtained a quite good fit. This means that the concentration dependent stabilization of the GQs can be explained by a simple thermodynamic model described above.

Similar stabilization of GQs was observed by Risitano and Fox for oligos similar to Htel, but they did not fit any theoretical curve to their data^61^.

**Figure 5:**
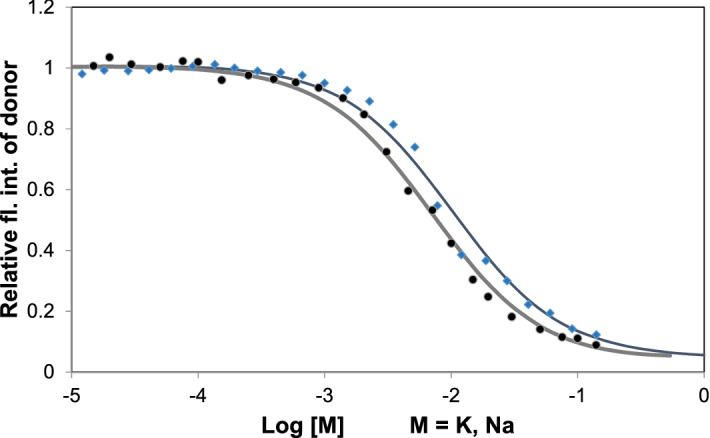
Relative donor fluorescence intensity of HepB as function of metal ion concentrations for Na^+^ (filled circle) and K^+^ (filled diamond) at 30 °C. Solid lines show the fitting to Eqs. (2) and (4).

The binding constants of Na^+^ and K^+^ were also measured. Figure 5 shows the folding of HepB as function of the concentration of Na^+^ and K^+^ at 30 °C (The spectra can be seen in Supplementary Fig. S4). The donor intensity decreases at around the dissociation constant (*K*_d_). The lines show the fit of Eqs. (2)–(5). The dissociation constants determined from the fit are 10.2 mM and 7.1 mM for K^+^ and Na^+^ respectively. The smaller dissociation constant of Na^+^ indicates a tighter binding to the GQ resulting in a higher temperature stability e.g. in *T*_m_ value. This means the titration results of HepB with K^+^ and Na^+^ ions are consistent with the temperature stability data.

### Binding of ligands to HepB

GQs started attracting attention of researchers, when their presence in the telomere region was proven. Several small molecules have been developed and investigated, which can stabilize the human telomere GQ. We have chosen three of them to see whether these can be used in case of our viral GQ. We investigated the three most important representative ligands: TMPyP4, BRACO19, PhenDC3.

### Influence of ligands on the temperature stability

All of the ligands increased the temperature stability of HepB. This was measured by fluorescence experiments. The ligands were added in twofold excess. TmpyP4 and PhenDC3 increased the unfolding temperature of HepB_FRET by 23 °C and 24 °C respectively. On the contrary, BRACO19 had a slight stabilization effect of 5 °C. This means all the studied ligands can bind the HepB oligo, and their binding stabilizes the folded structure. Our 5 °C stabilizing effect in case of BRACO19 is in agreement with the similar data in the literature. Majee et al. found stabilization effects between 3.6 and 13.1 °C for BRACO19 and on several GQs found in the genome of the Zika virus^62^.

### Determination of the dissociation constant of the ligands

**Figure 6:**
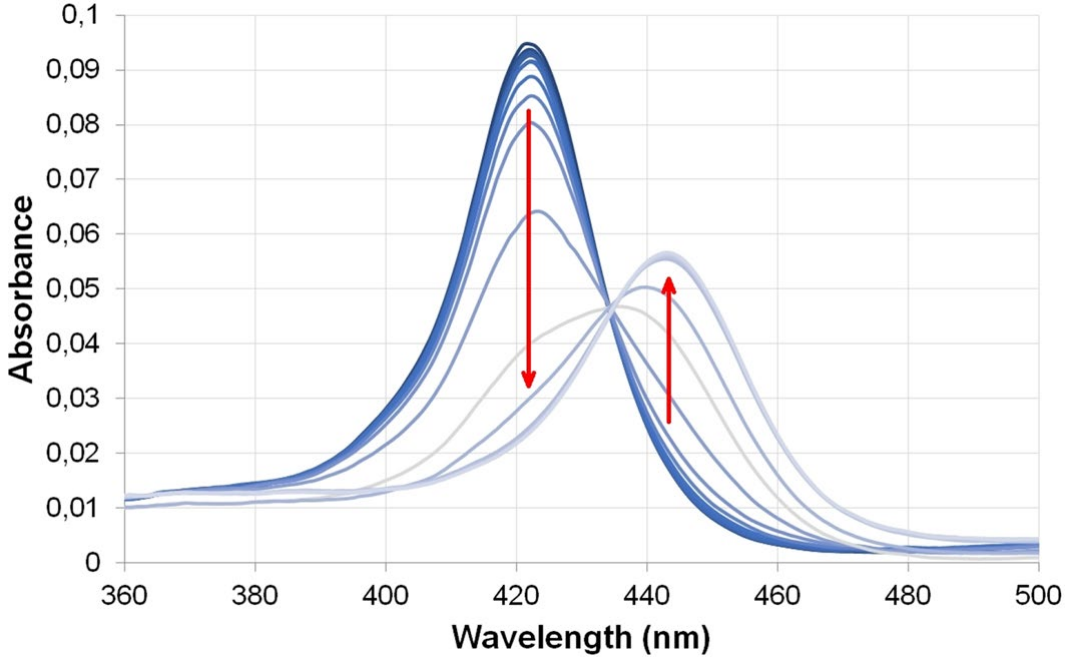
Absorbance spectra of TMPyP4 (0.5 μM) and HepB at concentrations from 10 nM to 10 μM. In 100 mM K-phosphate buffer pH 7.4. Arrows show the spectral changes due to increasing HepB concentration. The curves are corrected for the dilution of TMPyP4.

Titration of TMPyP4 with HepB was performed at different TMPyP4 concentrations. Both absorption and fluorescence spectroscopies were used. Figure 6 shows the absorption spectra of TMPyP4 at different HepB concentrations. Binding of HepB causes a red shift of 21 nm of the Soret band (from 422 to 443 nm) and 40% hipochromicity. Similar bathochromic shift has been observed when TMPyP4 bound to other GQs. Nagesh et al.^41^ observed 18 nm red shift and 60% hypochromicity for the Bcl-2 promoter GQ.

The appearance of the isobestic point in the plot of absorption spectra shows the two state character of the binding. These results suggest that the stoichiometry of the binding of TMPyP4 to HepB is 1:1. The same result can be obtained from the Job plot (Fig. S5). Unfortunately, similar experiments could not be performed for BRACO19 and PhenDC3, since their spectra overlap with that of the DNA oligo.

Since the binding constants of the oligos are quite low, they were determined using fluorescence spectroscopy. The ligands were excited at 420 nm, 285 nm and 290 nm for TMPyP4, BRACO19 and PhenDC3 respectively. Supplementary Figs. S6–S8 show the fluorescence emission spectra of BRACO19, PhenDC3 and TMPyP4 at different HepB concentrations. The bound molecules have significantly lower fluorescence intensity, which allows determining the bound fraction. The emission intensity changes were fitted to the Eqs. (2)–(5). The dissociation constant obtained for the three ligands are summarized in Table 2. As it can be seen, all the ligands bind to HepB_FRET.

**Table 2:** Dissociation constants of the studied ligands to HepB.

Ligand	*K*_d_ (M)
TMPyP4	(2.5 ± 0.6) × 10^–9^
BRACO19	(4 ± 1) × 10^–8^
PhenDC3	< 10^–6^

Le et al. Obtained K_d_ values of 0.21, 0.31 and 0.15 μM for binding of TMPyP4 to NRAS, c-MYC, and Htel sequences respectively. Nagesh et al. investigated the Bcl-2 promoter GQ and its mutants. TMPyP4 binds to these at two sites with dissociation constants of 10^–7^ M and 10^–5^ M^41^. Wei et al. studied the AG_3_(T_2_AG_3_)_3_ oligo, which is a section of the human telomere sequence. It binds TMPyP4 depending its conformation (parallel or hybrid) with the first binding constant of 2.26 × 10^8^ and 4.42 × 10^8^ M respectively^63^. These results compared with our values suggest, that TMPyP4 binds very strongly to HepB GQ, its affinity is similar or even higher than that of measured in case of human telomere GQ.

### Binding of ligand to unlabeled HepB

The above results show clearly that all the three ligands bind to the fluorescent labeled HepB. In order to prove that the fluorescence labeling does not considerably influence the binding, we used the competition^64^ assay described by Luo et al. This assay can prove that the binding is not restricted to the fluorescently labeled oligo, but the non-labeled oligos can also bind the three ligands we investigated. The method was slightly modified. Instead of investigating the binding of PhenDC3 to different oligos, we measured the binding of different ligands to the same oligo. The main point of the method is the following. Thermal unfolding curves are measured for three solutions: 1. oligo labeled by a FRET pair. 2. The same solution with the ligand. 3. The previous solution together with excess of unlabeled oligo. The binding of a ligand is expected to increase the stability of the oligo, which is measured as an increased unfolding temperature (*T*_m_). *T*_m_ returns to its original value (or close to it) if the excess unlabeled oligo will bind the ligand. If only the labeled oligo binds the ligand, and it does not bind the unlabeled, the third sample shows the same *T*_m_ as the second one. The *S*-factor defined by Luo et al. as *S* = (*T*_m3_ − *T*_m1_)/(*T*_m2_ − *T*_m1_) is close to zero if the unlabeled oligo binds the ligand, while it is close to 1 if only the FRET labeled oligo can bind the ligand (The indices of *T*_m_ in the definition of *S* refer to the solutions described above).

**Figure 7:**
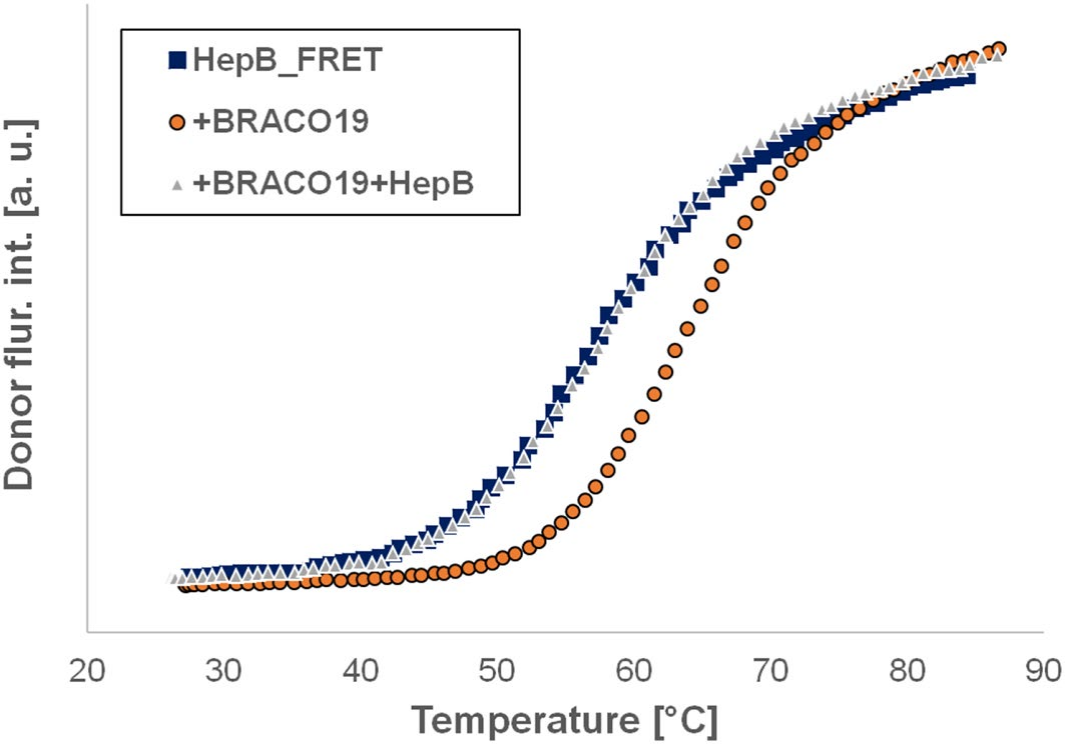
Fluorescence intensity of HepB_FRET (filled square), HepB_FRET + BRACO19 (filled circle), andHepB_FRET + BRACO19 + excess (nonlabeled) HepB (open triangle).

Figure 7 shows the three unfolding curves in case of BRACO19 (The spectra can be seen in Supplementary Fig. S9). As it can be seen the unlabeled oligo captured all the ligands, and the FRET labeled oligo showed the same fluorescence intensity profile as without BRACO19. Same experiments were performed with PhenDC3 and TMPyP4 too. The unfolding curves for PhenDC3 and TMPyP4 are shown in Figs. S10 and S11. Table 3 shows the temperature increases compared to the sole HepB_FRET solution, and also the calculated S values. It can be seen, that HepB binds all the three oligos we studied. In case of TMPyP4 and PhenDC3 an interesting effect has been observed: they bind to both the labeled and unlabeled GQ with high affinity. Normally, during the preparation of the third solution we added the unlabeled oligo in the last step. In this case we obtained a high S value, which indicated, that the ligands stay bound to the FRET labeled GQ, and do not switch for the unlabeled one. The experiments were repeated adding the labeled HepB in the last step; we obtained no increase of *T*_m_ compared to the first sample (when only the labeled HepB was present). This indicates, that both the labeled and unlabeled oligo bind TMPyP4 strongly. In case of BRACO19, the unlabeled oligo binds it better than the labeled.

**Table 3:** The stabilizing effect of the three ligands on HepB.

Ligand	*T*_m2_ − *T*_m1_ (°C)	*T*_m3_ − *T*_m1_ (°C)	S
PhenDC3	24	3	0.12
PhenDC3*	24	0	0.0
BRACO19	5	0.5	0.10
TMPyP4	23	14	0.61
TMPyP4*	23	1.5	0.07

Results of the competition assay. (Modified from Ref.^64^). *T*_m2_ − *T*_m1_ = increase of HepB_FRET when adding the ligand in twofold excess. *T*_m3_ − *T*_m1_ = increase of the transition midpoint when both the ligand and the unlabeled HepB were added. *S* = (*T*_m3_ − *T*_m1_)/(*T*_m2_ − *T*_m1_). First the ligand then the unlabeled HepB was added except of cases marked by *, where the unlabeled oligo was added first.

## Conclusion

All the studied metal ions (Na^+^, K^+^, Li^+^, and Rb^+^) induce GQ structure in the HepB sequence in the genome of the Hepatitis B virus. HepB shows a hybrid form in presence of K^+^, but all the other ions induce parallel structure. Higher concentrations of metal ions increase the unfolding temperature.

Study of three ligands designed for GQ targeting (TMPyP4, BRACO19 and PhenDC3) showed clearly their binding to HepB and to its fluorescently labeled variant. The binding to the unlabeled HepB was proven by the competitive assay. Additionally, the above results show an increased stability of the ligand bound GQs. The stabilization was higher than 20 °C for TMPyP4 and PhenDC3, while it was considerably lower for BRACO19.

Binding of the TMPyP4 and PhendDC3 to this viral GQ might have important medical relevance. These ligands, which were developed for cancer treatment, could have a potential role in the fight against the HepB virus. This hypothesis should however be confirmed by several further studies, but we believe this might be a promising new perspective.

## Supplementary Information

Supplementary Figures.
