# Supplementary material for: Characterization of a G-quadruplex from hepatitis B virus and its stabilization by binding TMPyP4, BRACO19 and PhenDC3
Source: Sci Rep. 2021 Dec 1;11:23243. doi: 10.1038/s41598-021-02689-y (PMC8636512; doi:10.1038/s41598-021-02689-y)
Supplement: Supplementary file 1 — Supplementary Figures. [file 41598_2021_2689_MOESM1_ESM.docx]

Supplementary Material for:

**Characterization of a G-quadruplex from hepatitis B virus and its stabilization by binding TMPyP4, BRACO19 and PhenDC3**

Orsolya Réka Molnár^1, *^, András Végh^1,2, *^, Judit Somkuti^1^ and László Smeller^1, **^

^1^ Department of Biophysics and Radiation Biology, Semmelweis University, Budapest, 1094, Hungary

^2^ Department of Ophthalmology, Semmelweis University, Budapest, 1085, Hungary

* These authors contributed the research equally

** To whom correspondence should be addressed. Tel: +36 1 459 1500; Fax: +36 1 266 6656; Email: smeller.laszlo@med.semmelweis-univ.hu

Figure S1:


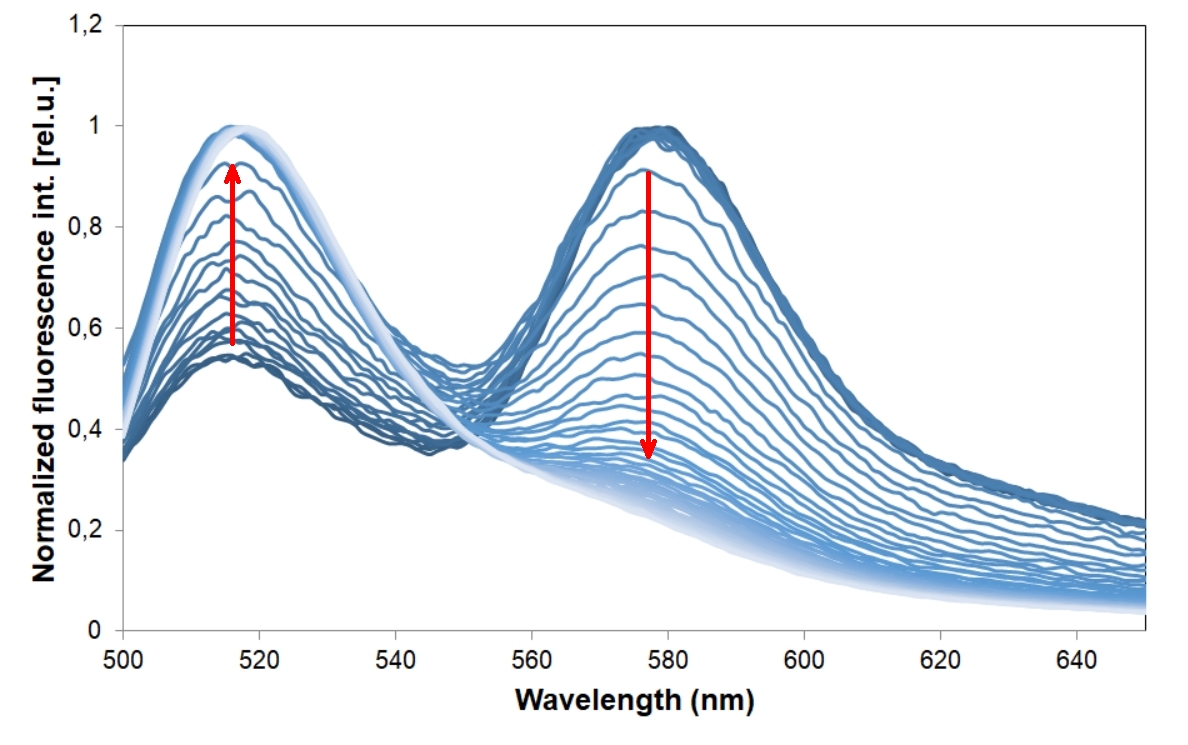


Figure S1: Fluorescence spectra of HepB labeled by a FRET pair of FAM and TAMRA in K‑phosphate buffer containing 140 mM K^+^ ion at temperatures from 30 °C to 90 °C. The darkest spectrum was taken at 30 °C, the brightest one at 90 °C. The arrows show the direction of the spectral changes during heating.

Figure S2


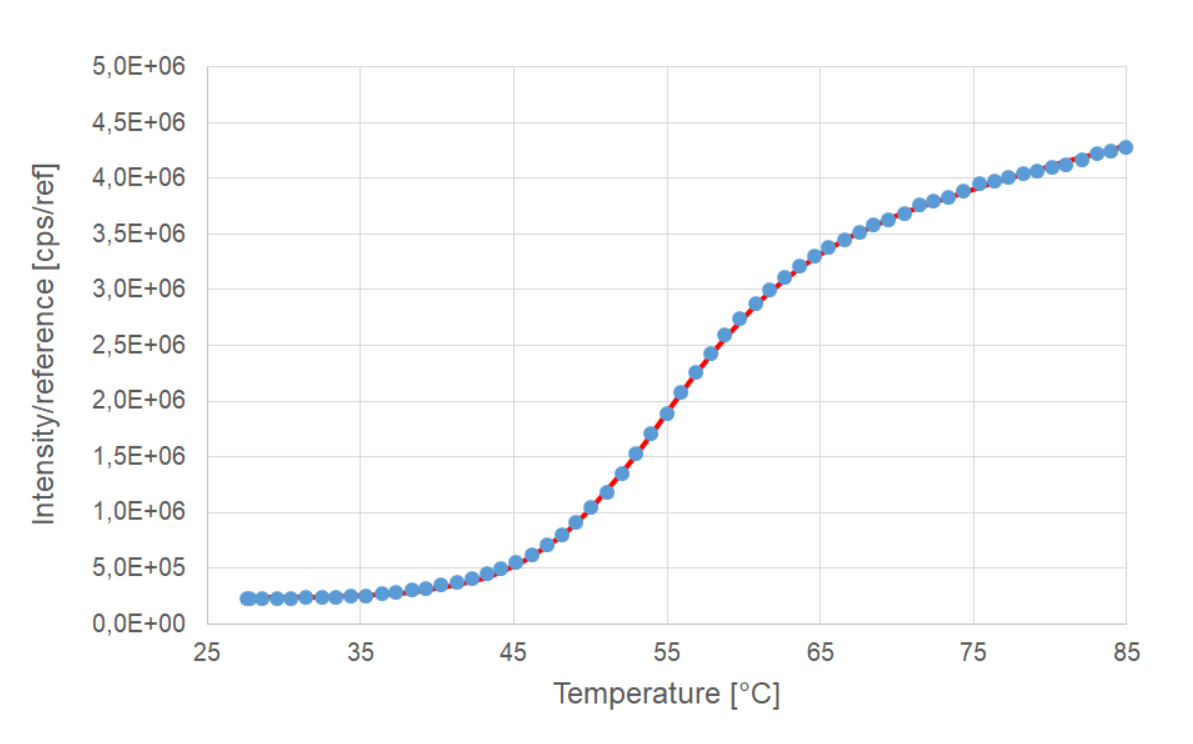


**b**

**a**


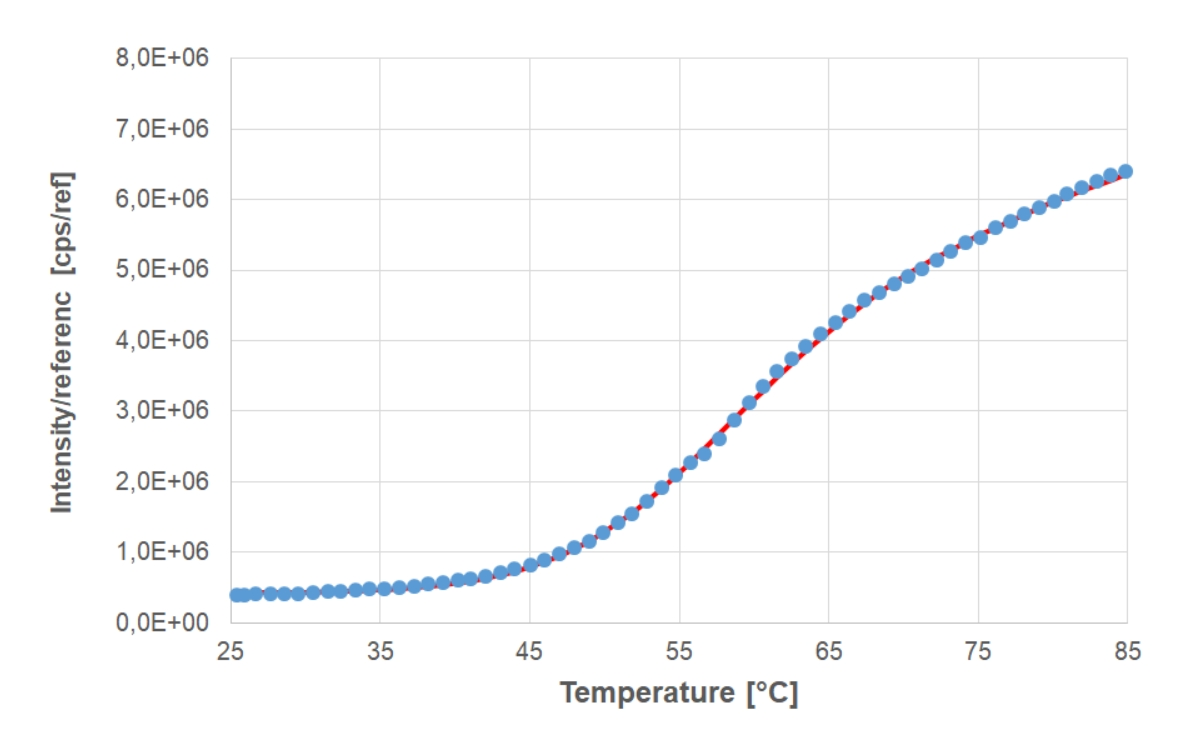


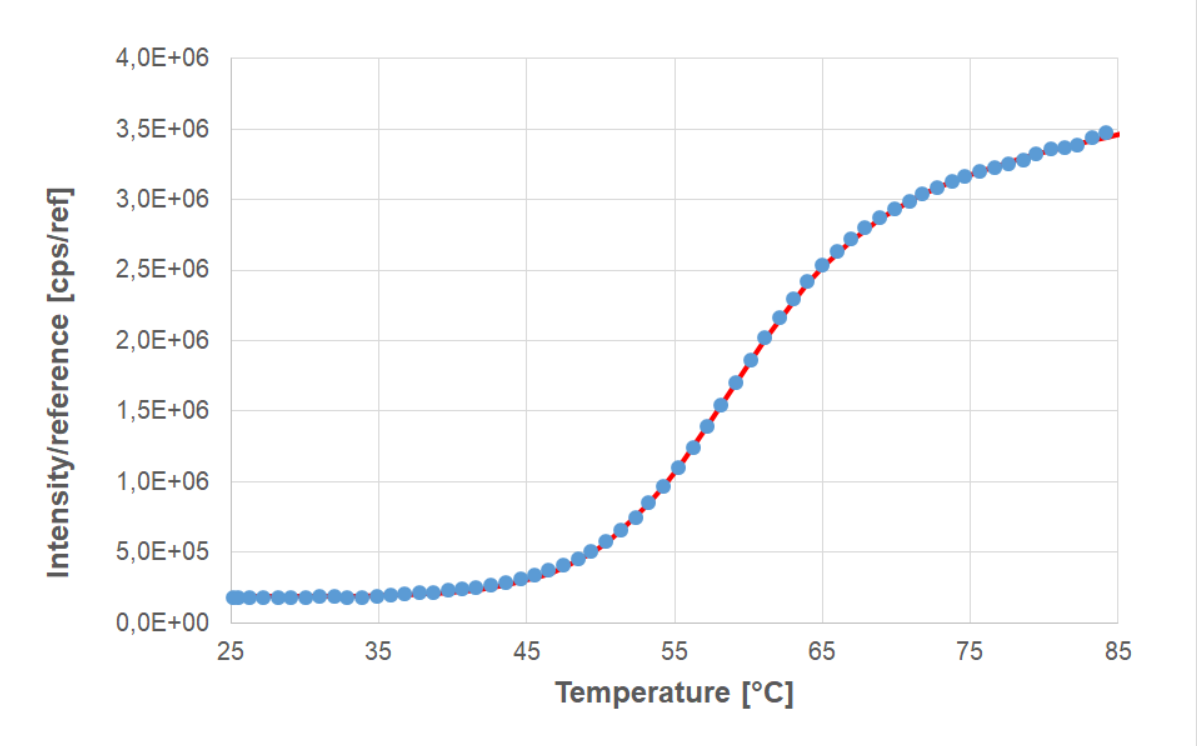


**d**

**c**


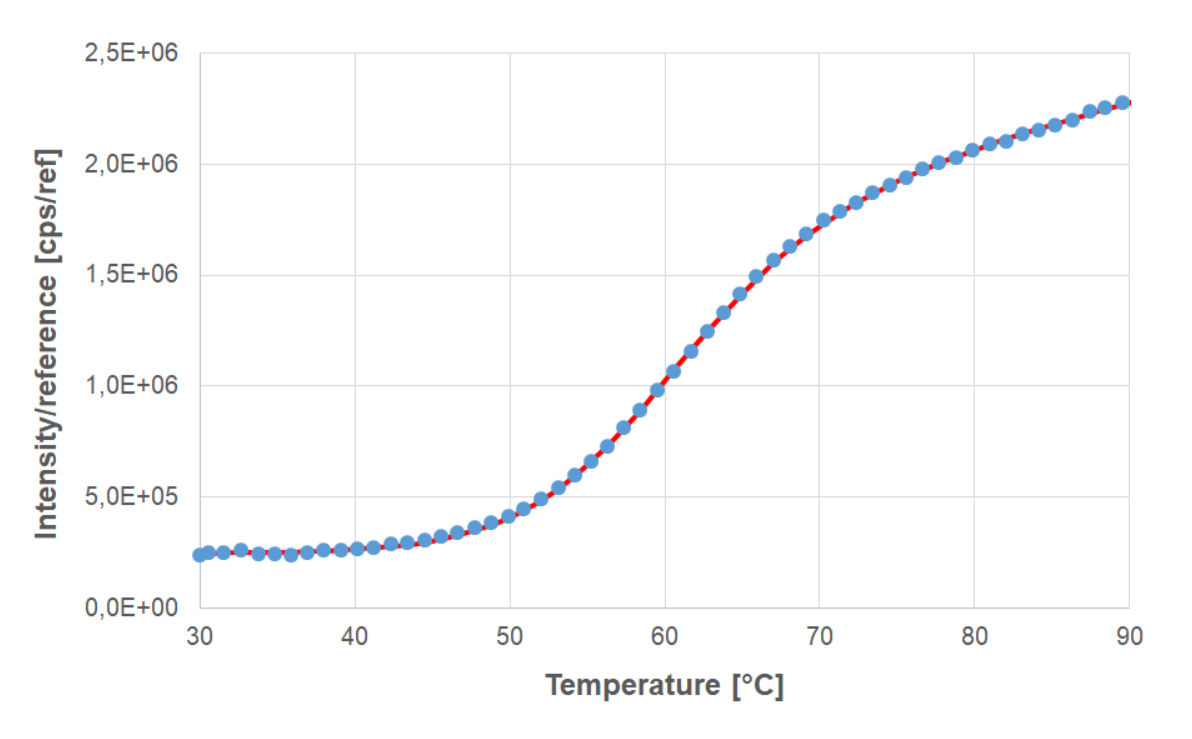


Figure S2: Donor fluorescence intensity of HEPB_FRET as function of the temperature at different Na^+^ concentrations: 100 mM **(a)**, 140 mM **(b)**, 175 mM **(c)** and 260 mM **(d)**. The fluorescence intensity is divided by the signal of the reference diode of the spectrometer. The transition temperatures are 54.0, 56.6, 58.2 and 59.7 °C respectively.

Figure S3

**a**


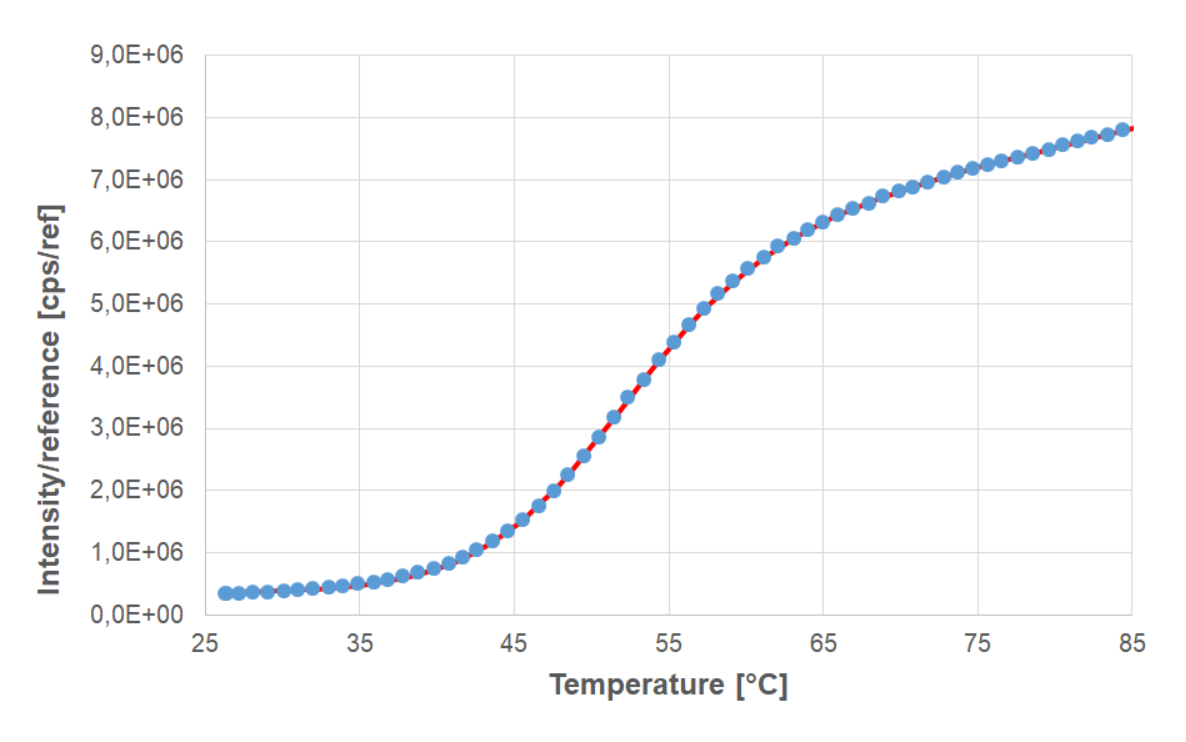


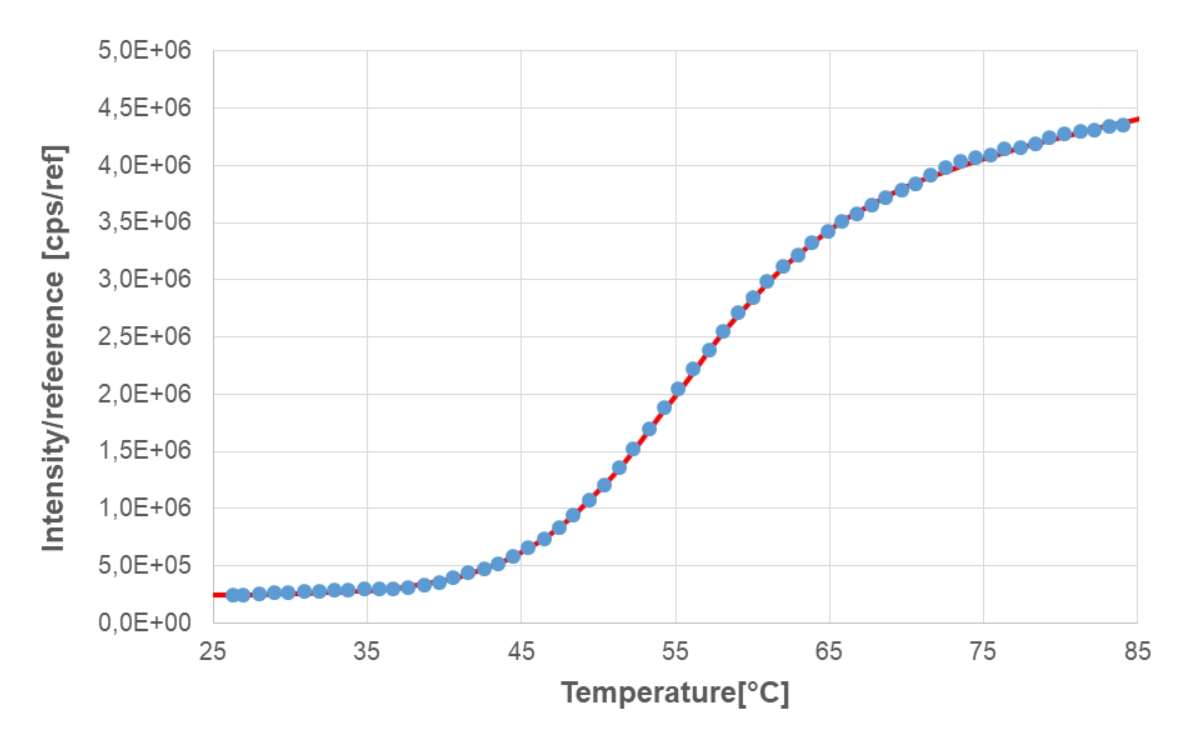


**b**


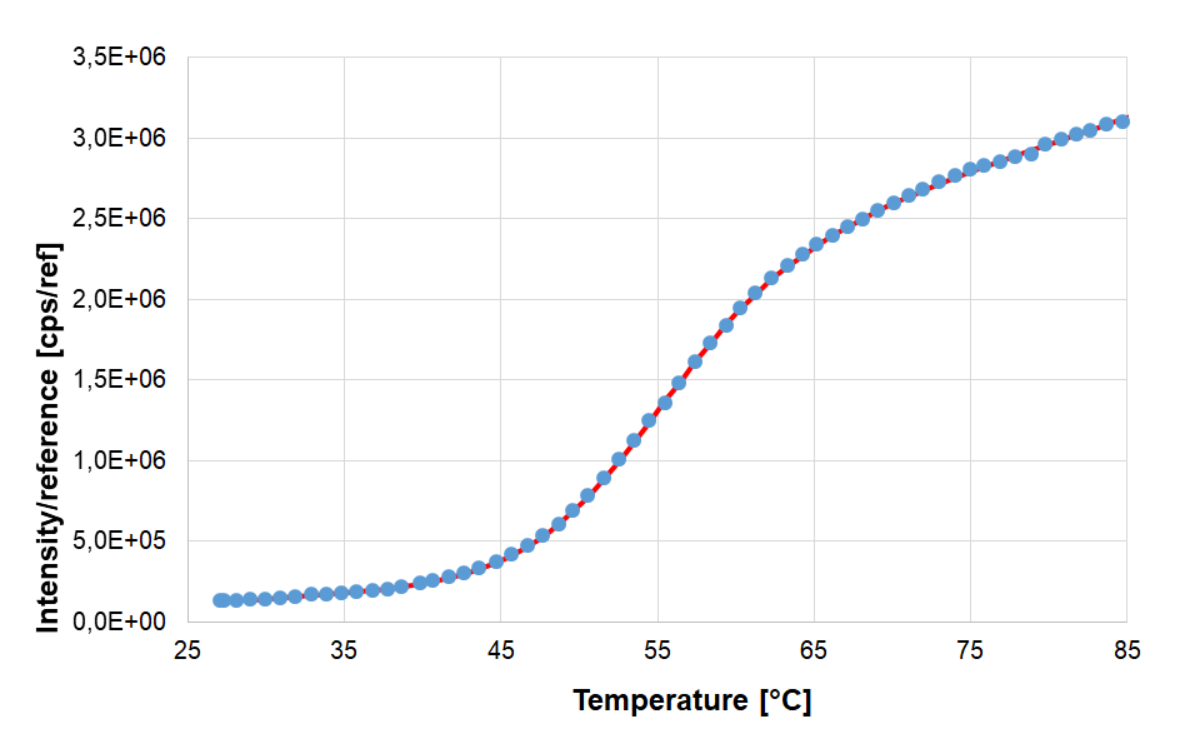


**c**


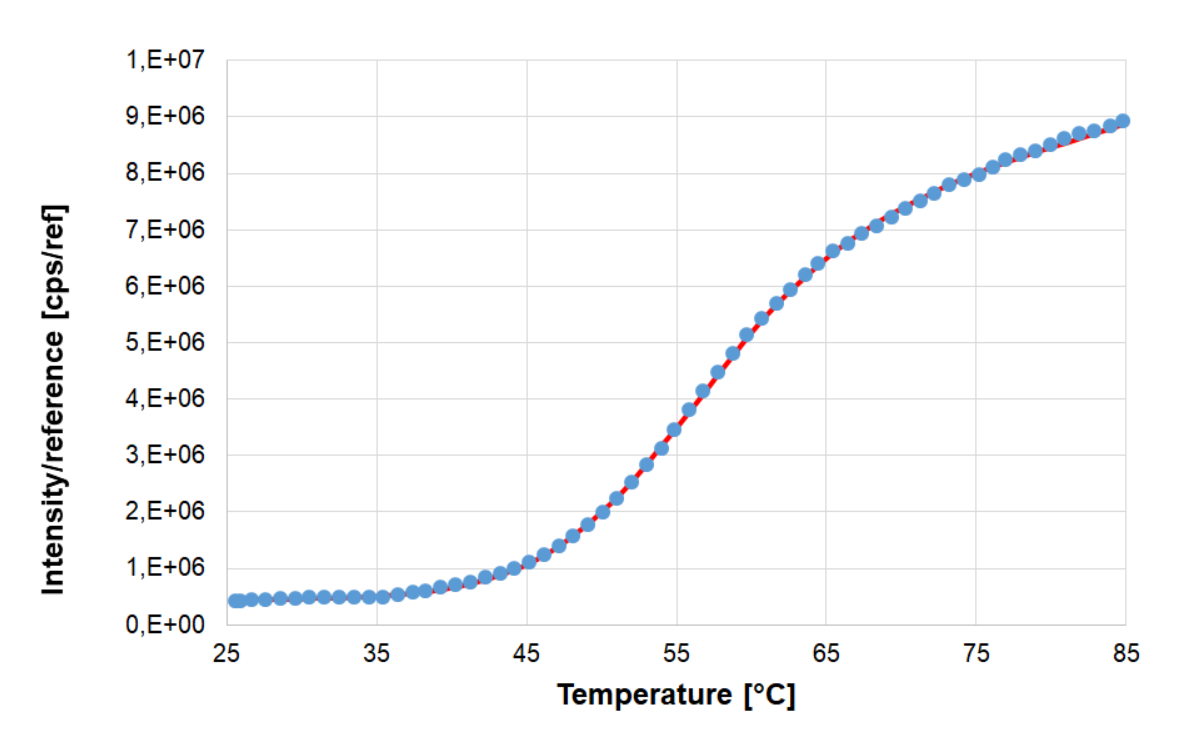


**d**


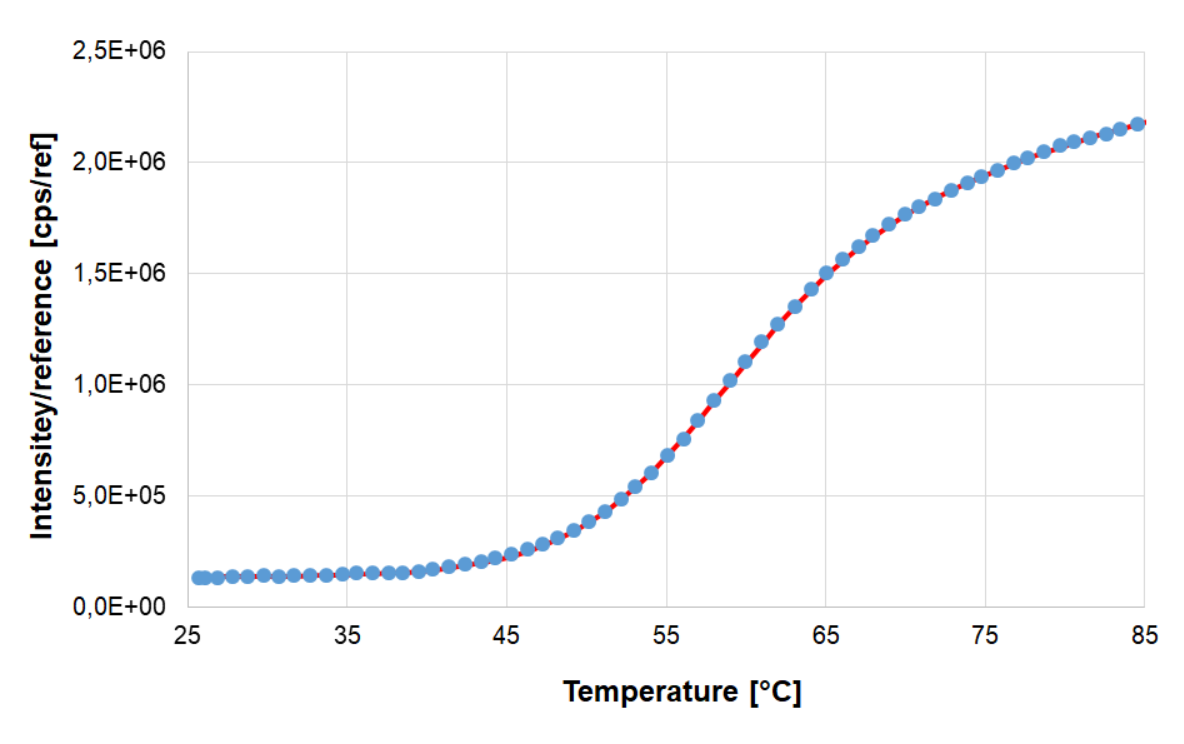
Figure S3: Donor fluorescence intensity of HEPB_FRET as function of the temperature at different K^+^ concentrations: 100 mM **(a)**, 140 mM **(b)**, 170 mM **(c)**, 175 mM **(d)** and 260 mM **(e)**. The second experiment at 140 mM is presented in Figure 1. The fluorescence intensity is divided by the signal of the reference diode of the spectrometer. The transition temperatures are 51.4, 54.5, 54.4, 55.4 and 58.1 °C respectively.

**e**

Figure S4:

**a**


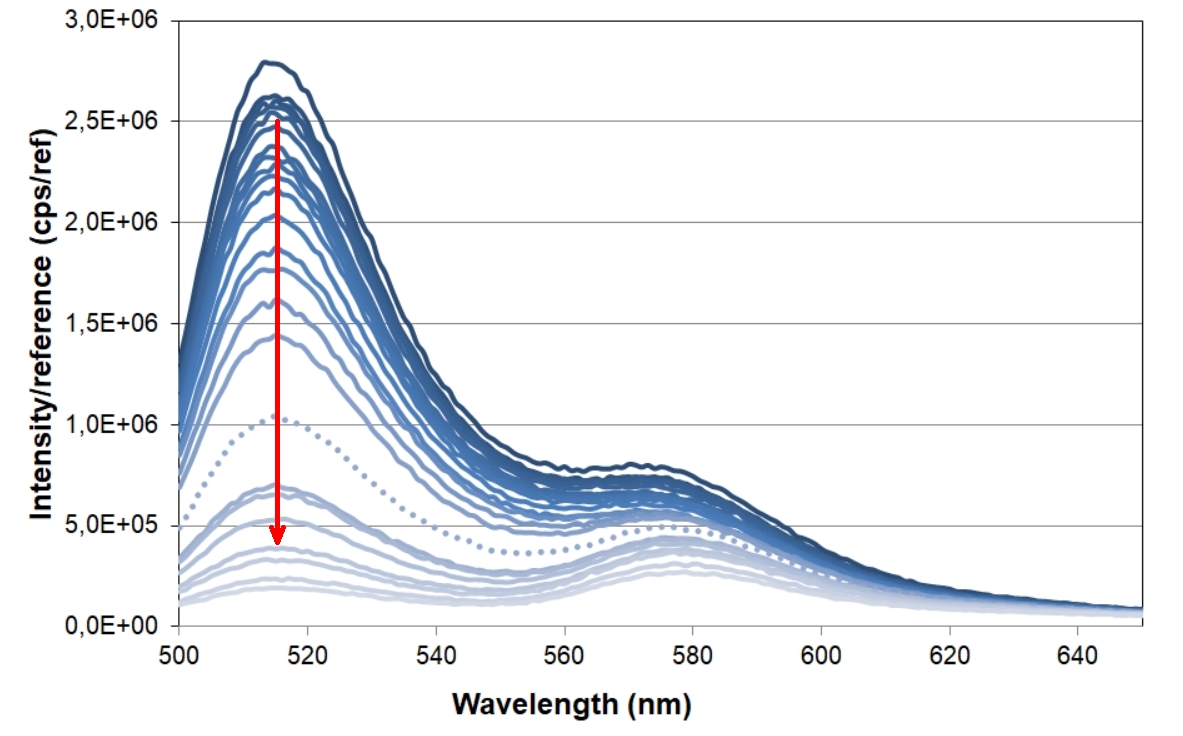


**b**


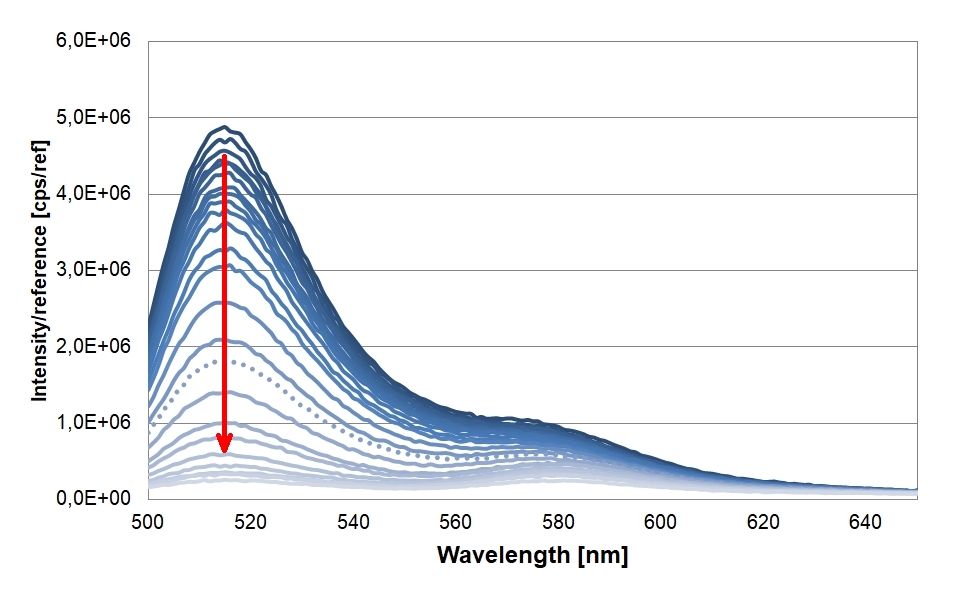


Figure S4: **(a)** Fluorescence spectra of HEPB_FRET at various KCl concentrations. The concentration values are: 12.0 (darkest spectrum), 18.0, 28.0, 40.9, 61.1, 90.6, 135, 194, 295, 450, 666, 997 μM, 1.51, 2.26, 3.49, 5.17, 7.80 (dotted line), 12.0, 21.3, 18.8, 27.7, 40.7, 60.6, 90.4 and 140 mM. The arrow shows the direction of the spectral changes during increase of the KCl concentration.
**(b)** Fluorescence spectra of HEPB_FRET at various NaCl concentrations. The concentration values are: 14.9 (darkest spectrum), 19.9, 29.7, 50.1, 75.6, 99.8, 153, 250, 395, 592, 898 μM, 1.40, 2.05, 3.08, 4.59, 7.11 (dotted line), 10.1, 14.9, 19.6, 30.2, 50.6, 75.4, 100 and 140 mM. The initial decrease of the intensity is due to the dilution in both cases. The arrow shows the direction of the spectral changes during increase of the NaCl concentration.

Figure S5:


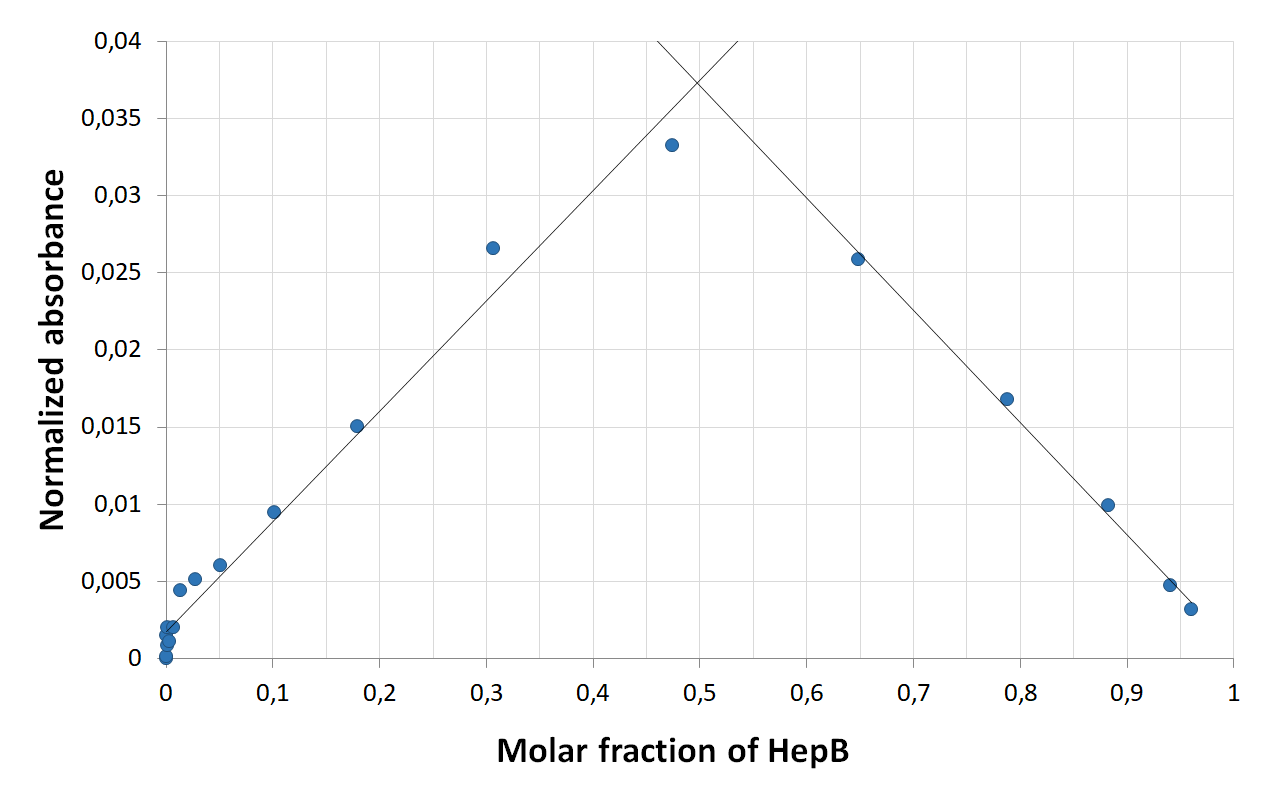


Figure S5. Job plot of the binding of TMPyP4 to HepB. The absorbance change at 443 nm was normalized taking into account the concentration changes during the measurement. Solid lines are fitted to the point below and above 0.5 molar fractions.

Figure S6:


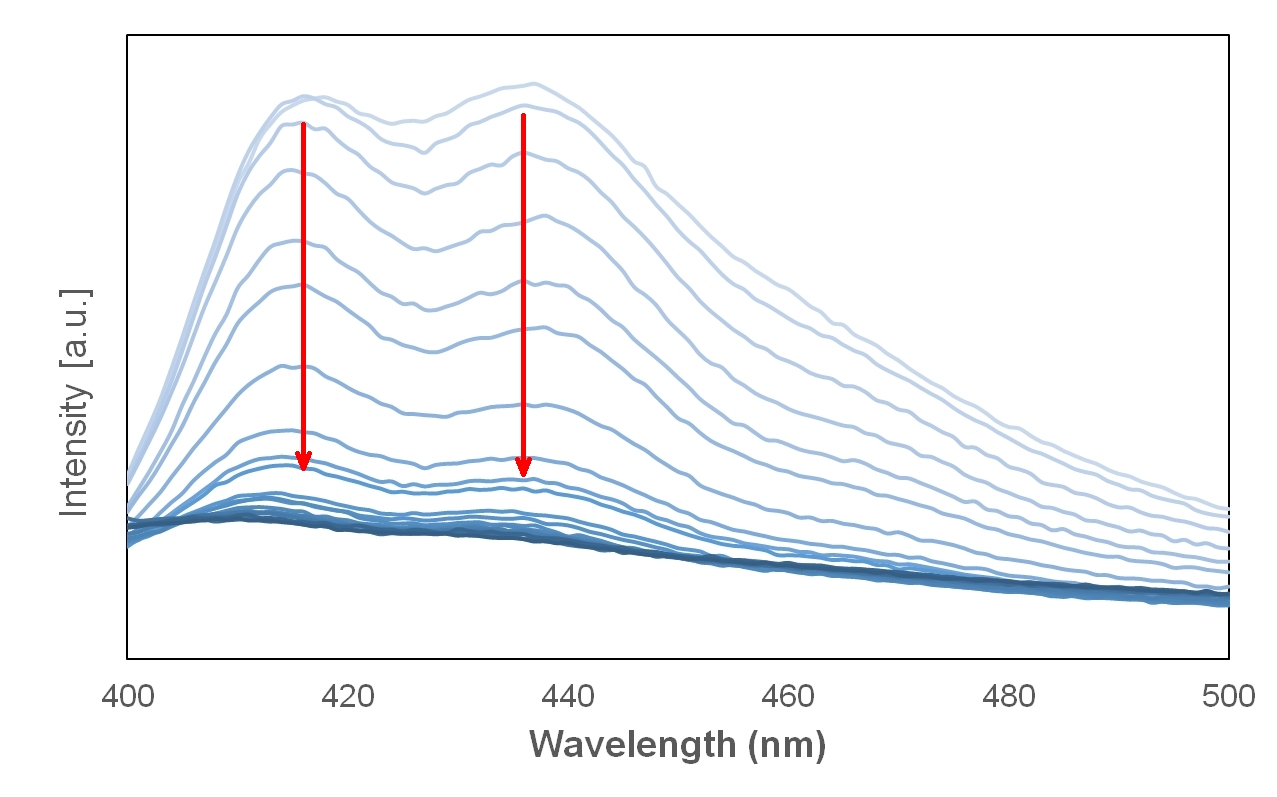


Figure S6: BRACO19 spectra in presence of increasing amount of (unlabeled) HepB. The HepB concentrations from top to bottom are: 0.01, 0.015, 0.023, 0.035, 0.051, 0.075, 0.12, 0.17, 0.25, 0.38, 0.60, 0.84, 1.2, 1.9, 2.9, 4.4, 6.4, 9.9, 15.5, 22, 33, 50 µM. Arrows show the direction of the spectral changes due to increasing HepB concentration.

Figure S7:


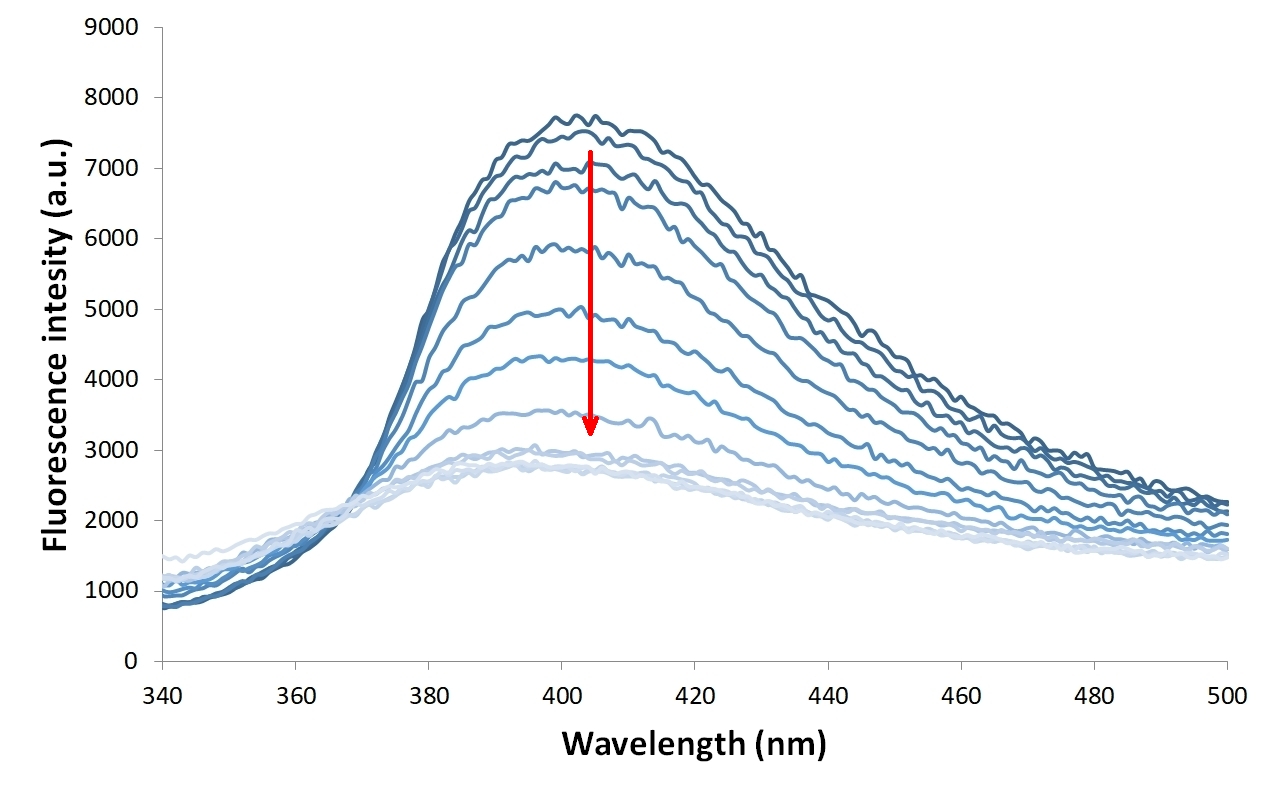


Figure 7: PhenDC3 spectra in presence of increasing amount of (unlabeled) HepB. The HepB concentrations from top to bottom are 0, 0.01, 0.022, 0.051, 0.075, 0.17, 0.6, 1.2, 6.4, 10, 15, 22 33, 50 µM. Arrow shows the direction of the spectral changes due to increasing HepB concentration.

Figure S8:


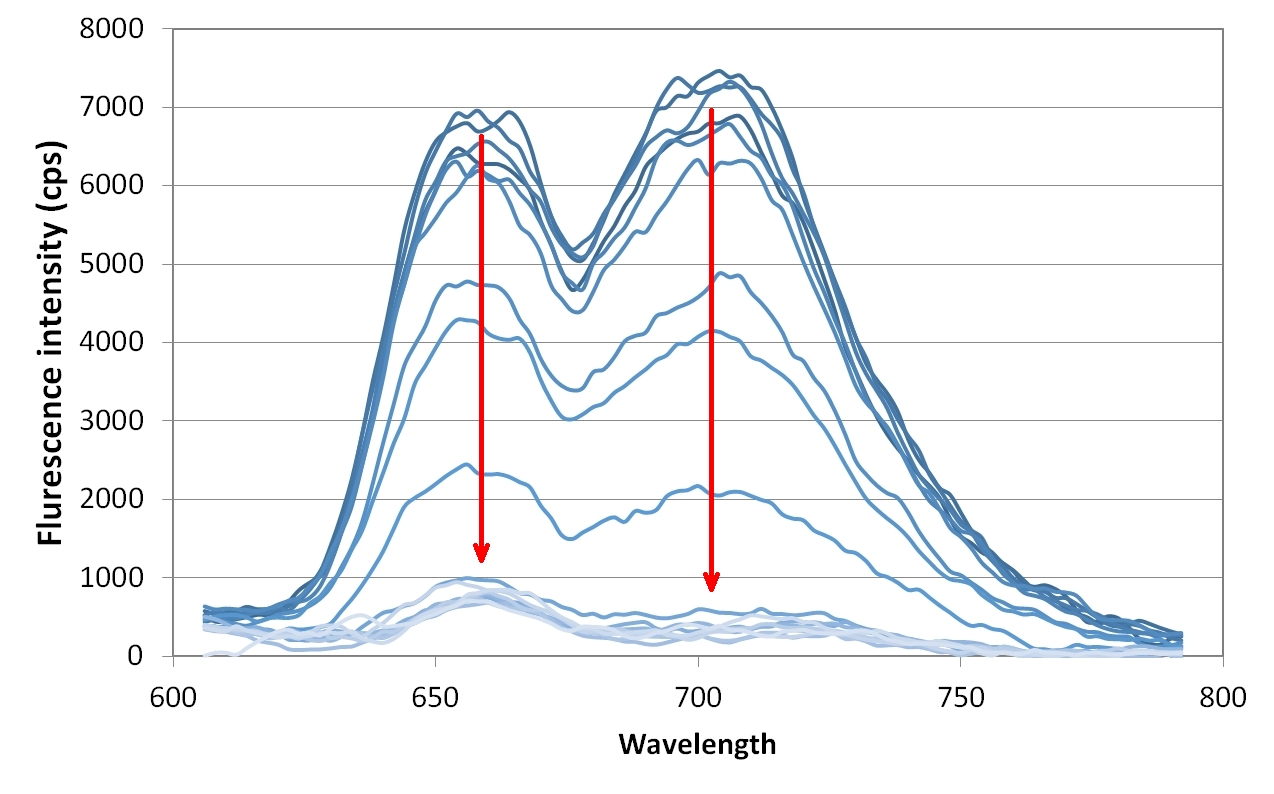


Fig. S8: TMPyP4 spectra in presence of increasing amount of (unlabeled) HepB. The HepB concentrations from top to bottom are 0.1, 1.2, 0.4, 0.8, 1.55, 3.2, 6.4, 12, 25, 50, 100, 200 800 nM , 1.6, 3.2, 6.5, 10 µM. Arrows show the direction of the spectral changes due to increasing HepB concentration.

Figure S9:


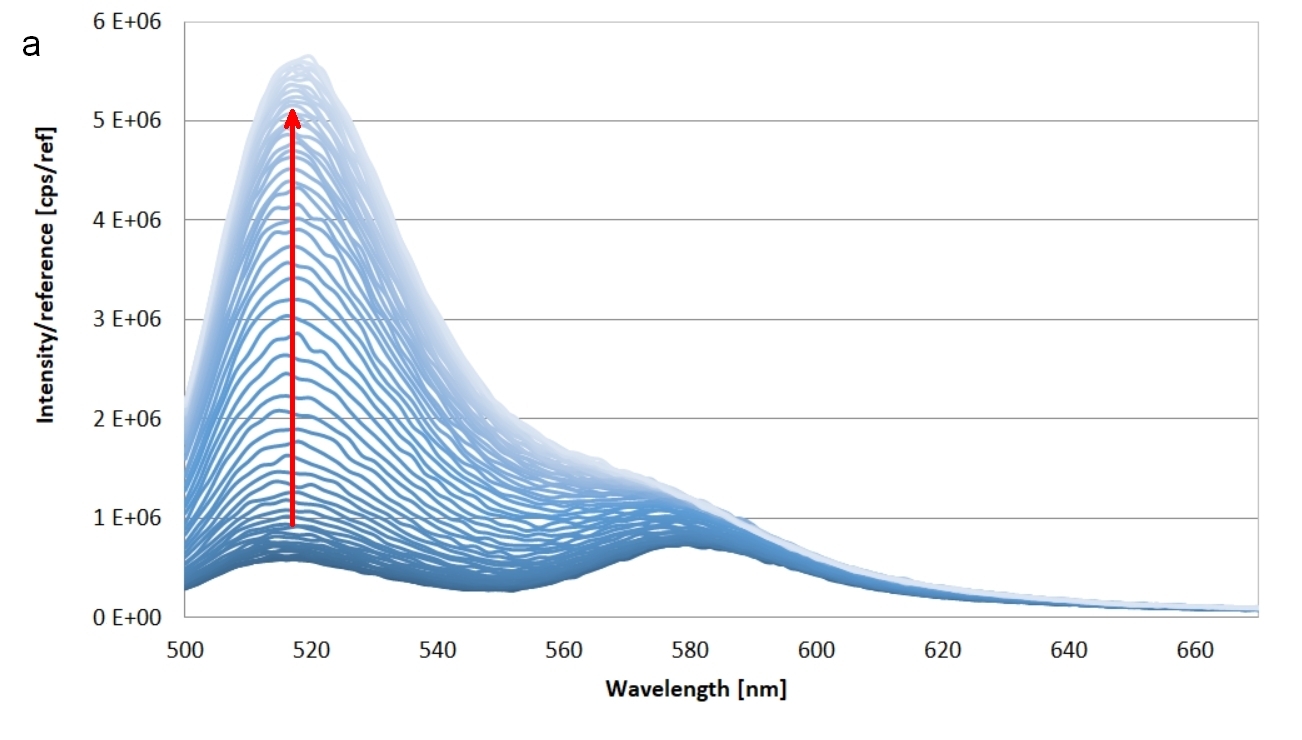


**a**

**
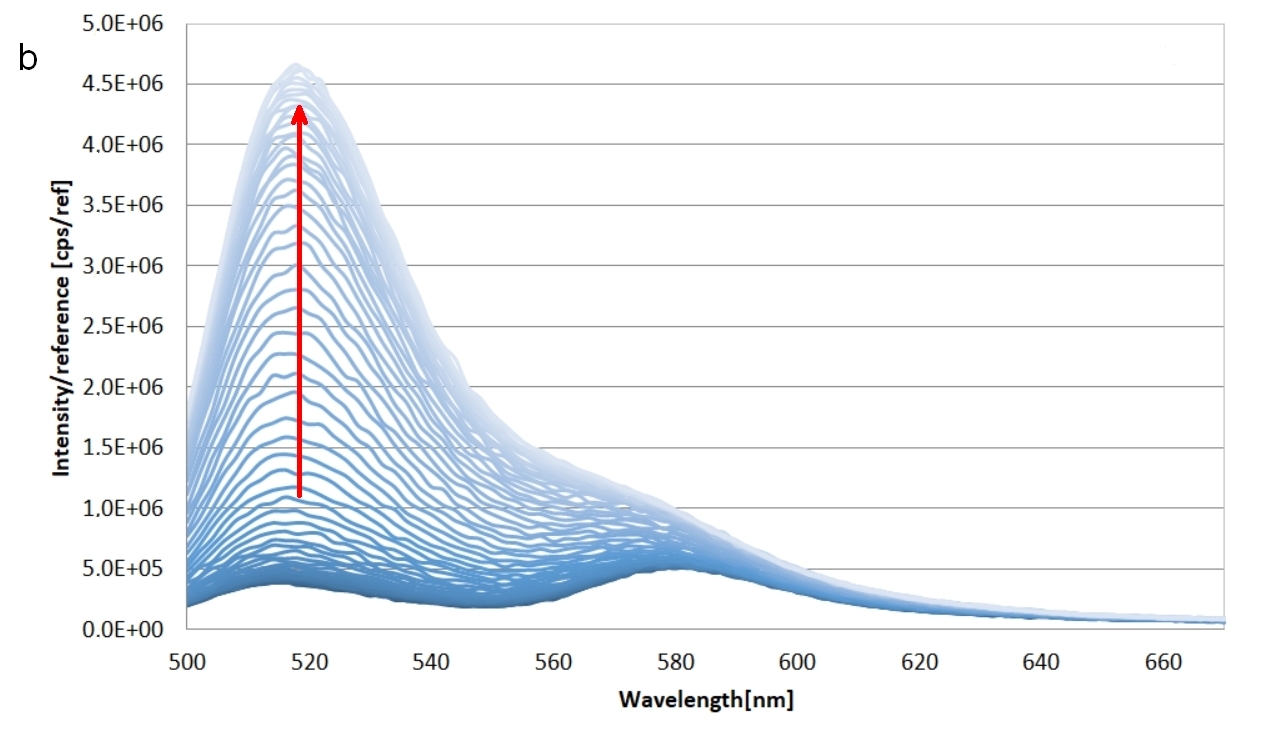
**

**b**


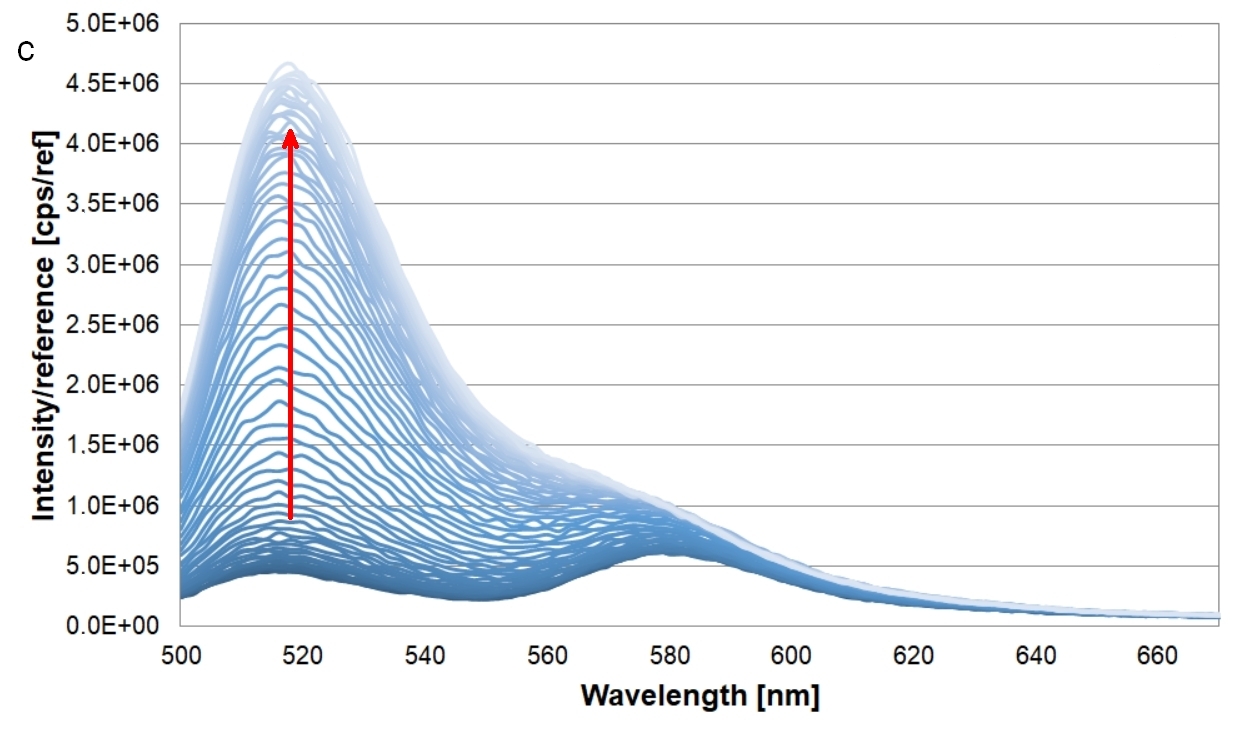


**c**

Figure S9: Fluorescence spectra of 1 μM HEPB_FRET **(a)**, 1 μM HEPB_FRET + 2μM BRACO19 **(b**), 1 μM HEPB_FRET + 2μM BRACO19 + 10 μM unlabeled HEBP **(c)**. Temperature increases from 30 °C (darkest spectrum) to 85 °C by 1 °C/spectrum. The arrow shows the spectral changes during temperature increase.

Figure S10:
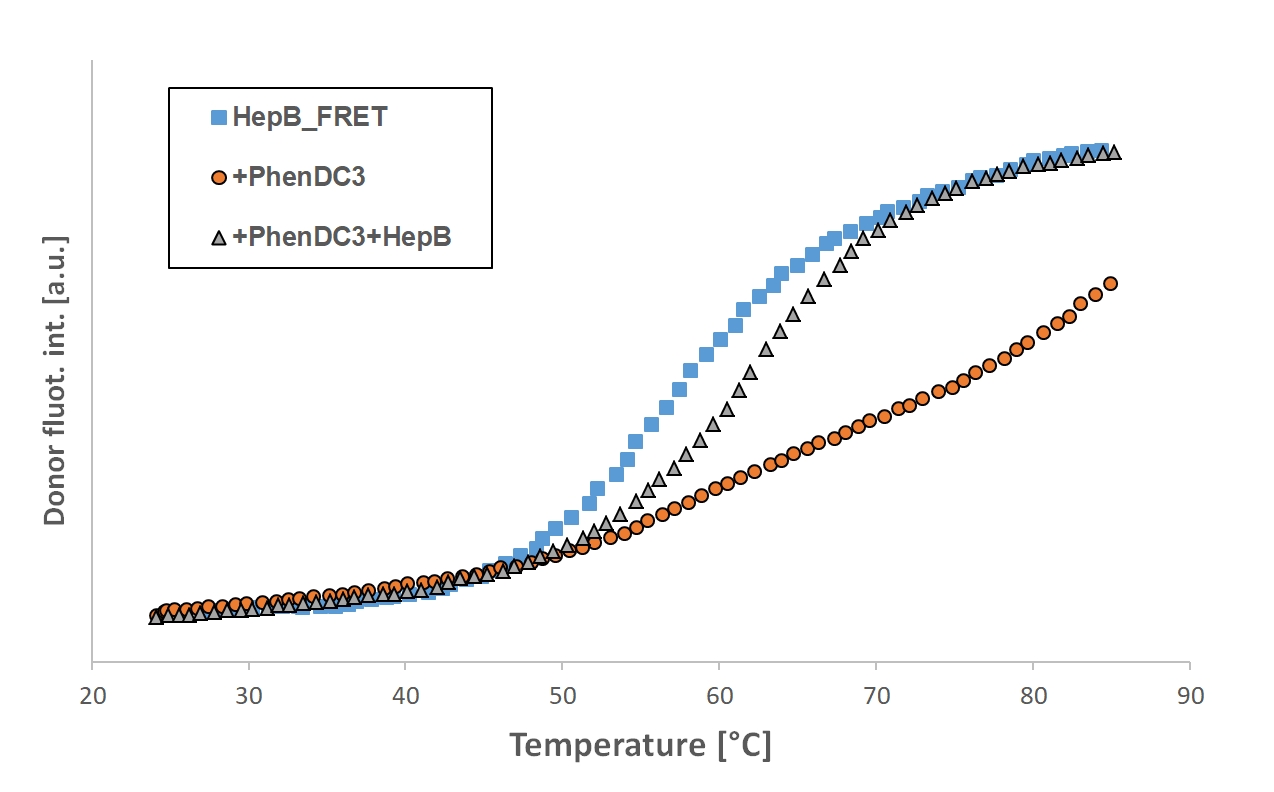


Figure 10: Fluorescence intensity of HepB_FRET (◼), HepB_FRET+PhenDC3 (•), and HepB_FRET+PhenDC3+excess (nonlabeled) HepB (Δ).

Figure S11:


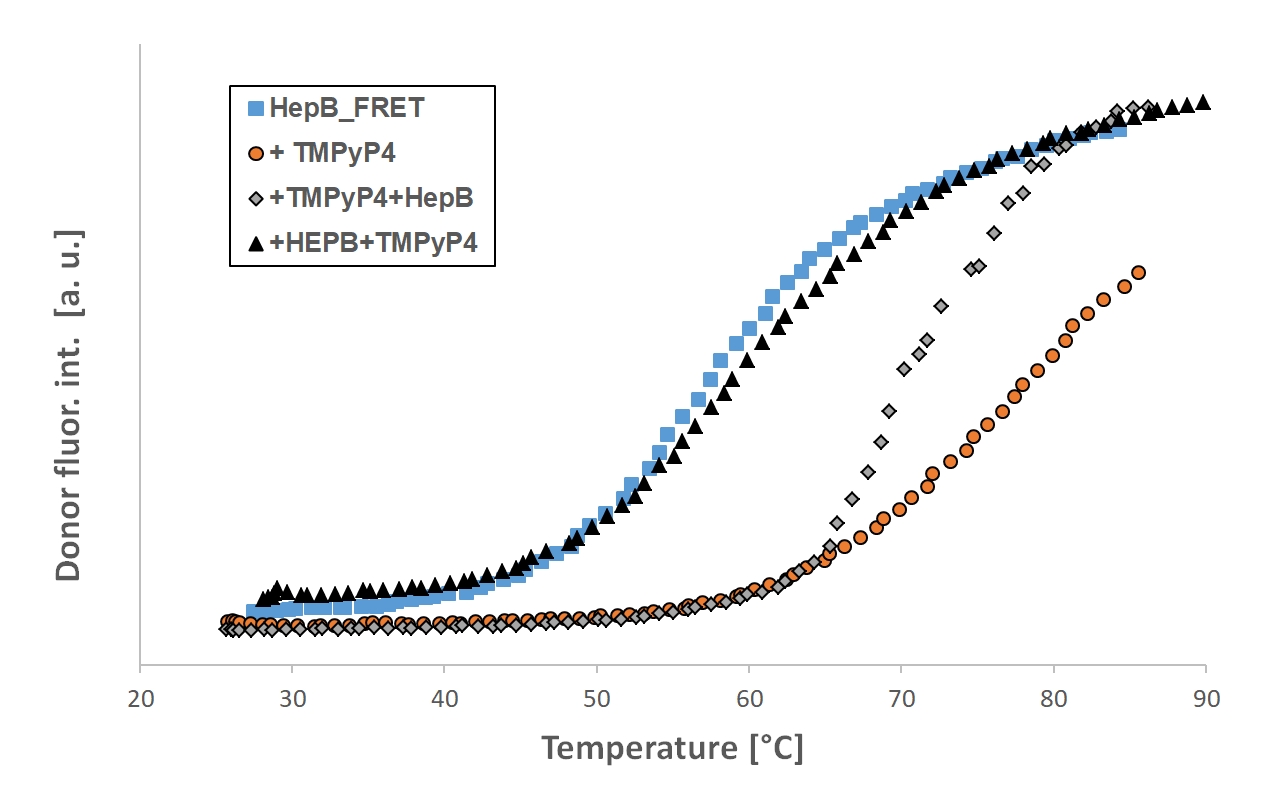


Figure S11. Fluorescence intensity of HepB_FRET (◼), HepB_FRET+TMPyP4 (•), HepB_FRET+ TMPyP4 +excess (nonlabeled) HepB (♦) + and HepB_FRET +excess (nonlabeled) HepB +BRACO19 (Δ).
